# The development of an anti-cancer peptide M1-21 targeting transcription factor FOXM1

**DOI:** 10.1186/s13578-023-01059-7

**Published:** 2023-06-21

**Authors:** Haojie Cheng, Jie Yuan, Chaozhu Pei, Min Ouyang, Huitong Bu, Yan Chen, Xiaoqin Huang, Zhenwang Zhang, Li Yu, Yongjun Tan

**Affiliations:** 1 State Key Laboratory of Chemo/Biosensing and Chemometrics, College of Biology, Hunan Engineering Research Center for Anticancer Targeted Protein Pharmaceuticals, Hunan University, 410082 Changsha, Hunan China; 2 Medicine Research Institute, Hubei Key Laboratory of Diabetes and Angiopathy, Xianning Medical College, Hubei University of Science and Technology, 437000 Xianning, Hubei China

**Keywords:** M1-21, D-retro-inverso (DRI) peptide, Transcription factor FOXM1, Interfering peptide, Cancer treatment

## Abstract

**Background:**

Transcription factor FOXM1 is a potential target for anti-cancer drug development. An interfering peptide M1-21, targeting FOXM1 and FOXM1-interacting proteins, is developed and its anti-cancer efficacy is evaluated.

**Methods:**

FOXM1 C-terminus-binding peptides are screened by *in silico* protocols from the peptide library of FOXM1 (1-138aa) and confirmed by cellular experiments. The selected peptide is synthesized into its D-retro-inverso (DRI) form by fusing a TAT cell-penetrating sequence. Anti-cancer activities are evaluated in vitro and in vivo with tumor-grafted nude mice, spontaneous breast cancer mice, and wild-type metastasis-tracing mice. Anti-cancer mechanisms are analyzed. Distribution and safety profiles in mice are evaluated.

**Results:**

With improved stability and cell inhibitory activity compared to the parent peptide, M1-21 binds to multiple regions of FOXM1 and interferes with protein-protein interactions between FOXM1 and its various known partner proteins, including PLK1, LIN9 and B-MYB of the MuvB complex, and β-catenin. Consequently, M1-21 inhibits FOXM1-related transcriptional activities and FOXM1-mediated nuclear importation of β-catenin and β-catenin transcriptional activities. M1-21 inhibits multiple types of cancer (20 µM in vitro or 30 mg/kg in vivo) by preventing proliferation, migration, and WNT signaling. Distribution and safety profiles of M1-21 are favorable (broad distribution and > 15 h stability in mice) and the tested non-severely toxic dose reaches 200 mg/kg in mice. M1-21 also has low hemolytic toxicity and immunogenicity in mice.

**Conclusions:**

M1-21 is a promising interfering peptide targeting FOXM1 for the development of anti-cancer drugs.

**Supplementary Information:**

The online version contains supplementary material available at 10.1186/s13578-023-01059-7.

## Introduction

Transcription factor FOXM1 is expressed in all embryonic tissues during development but only in adult tissues with a high proliferation index [1]. Based on clinical sample data, FOXM1 has been found to overexpress in multiple cancers and FOXM1 levels can predict disease diagnosis and prognosis [2]. As an oncoprotein, FOXM1 activates a series of key genes for cell cycle and mitosis to promote the malignant proliferation of cancer cells [3]. FOXM1 is also involved in promoting metastasis and invasion of cancer cells [4] and maintaining the characteristics of cancer stem cells [5, 6]. In terms of FOXM1-stimulating gene expression mechanisms, the FOXM1 DNA binding domain (DBD, 232-332aa) binds directly to its conserved RYAAAYA motif (FKH motif) in downstream gene promoters and thus activates their transcription [7]. FOXM1 also stimulates gene expression through an indirect DNA binding mechanism mediated by the MuvB complex [8, 9]. This complex consists of multiple subunits such as LIN9 and B-MYB and binds to the CHR (Cell cycle genes Homology Region) motif (TTTGAA or TTTAAA) in target gene promoters, and controls precisely timed transcription of the cell cycle [10]. FOXM1 is recruited by LIN9 and B-MYB of the MuvB complex via the FOXM1 N-terminal domain (1-138aa), thus enabling transcriptional activation of certain cell cycle genes without FOXM1 binding directly to DNA [8, 9]. FOXM1 transcriptional activities are induced by cell cycle-related protein kinases such as CDK1 [11] and PLK1 [12] through phosphorylation at multiple sites, particularly in the FOXM1 C-terminal domain (688-748aa). The phosphorylated FOXM1 then interacts with the CBP complex to induce transcription of target genes [11, 12]. In addition, FOXM1 can facilitate the nuclear import process of other cancer-related transcription factors through protein-protein interactions to boost the transcriptional activities of those transcription factors, evidenced by the findings that the nuclear importing of β-catenin or Smad3 is dependent on their interaction with FOXM1 for fully activating the classical WNT or TGF-β signaling pathway [13, 14].

FOXM1 is considered a potential target for anti-cancer drug development due to its multiple roles in stimulating cancer cells [15]. Our cancer gene therapy studies of multiple cancers with adenovirus-mediated knockdown of FOXM1 expression have supported this concept [16–18]. To disrupt the transcriptional activities of FOXM1, small molecule chemical compounds have been screened, including Thiostrepton [19], antibiotic Siomycin A [20], RCM-1 [21], and FDI-6 [22, 23], for abolishing the proliferation of cancer cells. Furthermore, cell-penetrating ARF26-44 peptide [24] derived from tumor suppressor protein p19ARF and 9R-P201 peptide [25] selected from a random dodecapeptide library against FOXM1 DBD can inhibit FOXM1 transcriptional activities in cancer cells. We have also selected a FOXM1 DBD-specific single-strand DNA aptamer to inhibit FOXM1 transcriptional functions in cancer cells [26]. PROTAC strategy has also been used to target FOXM1 protein through E3 ligase-mediated degradation [27]. However, commercial anti-cancer therapeutics targeting FOXM1 are not yet available.

Recently, we have obtained a new cell-penetrating protein reagent, M1-138, which is a recombinant FOXM1 N-terminal domain (1-138aa) fused with a nine arginine cell-penetrating peptide, to reduce the proliferation and migration abilities of cancer cells by targeting FOXM1 and FOXM1-interacting factor SMAD3 [28]. We have demonstrated that M1-138 binds to the FOXM1 C-terminal domain to prevent FOXM1-mediated transcriptional activation directly, and also competes with FOXM1 interaction with the MuvB complex to suppress FOXM1/MuvB-mediated gene activation. Therefore, M1-138 can attenuate FOXM1 transcriptional activities from both direct and indirect FOXM1-promoter binding mechanisms. Furthermore, M1-138 prevents nuclear importation of SMAD3 by interfering with the FOXM1:SMAD3 interaction, thus abolishing SMAD3 transcriptional activities. Treatment of M1-138 to xeno-grafted cancers in nude mouse models shows dramatic inhibition of tumorigenicity and cancer growth. Thus, M1-138 provides a basis for further screening of so-called interfering peptides for the development of anti-cancer drugs targeting FOXM1.

Interfering peptides have been considered a new class of drugs targeting protein-protein interactions that mediate many biological functions and are well recognized as promising therapeutic targets [29]. Because large and flat contact surfaces (missing features such as pockets or grooves) often mediate protein-protein interactions, peptides created to bind to these surfaces function better than small molecules to interfere with protein-protein interactions [30]. Moreover, the use of cell-penetrating peptides with the ability to transport different cargo becomes a promising option to improve intracellular peptide delivery [31]. At present, several anti-cancer interfering peptides, disrupting the interfaces of protein-protein interactions involved in proliferation, apoptosis, or inflammation, have been validated for cancer treatment in preclinical studies (e.g., the peptide derived from TCF4 N-terminus targeting the β-catenin:TCF interaction [32], the peptide derived from p73 DBD targeting the p53:p73 interaction [33], and the peptide derived from FOXO4 targeting the FOXO4:p53 interaction [34]) or in clinical trials (e.g., NSC745104 targeting the p53:HDM2 interaction [35] and XG-102 targeting the JNK:c-Jun interaction [36]). All interfering peptides have been rationally designed from natural sequences that mediate protein-protein interactions in proteins. In addition, *in silico* approaches have been used to optimize peptide sequences to improve their sensitivity and specificity to target proteins [37], and a series of chemical optimization approaches, including D-retro-inverso peptide [38], have been developed to enhance peptide stability by facilitating their resistance to protease degradation. Based on the fact that they are expressed in virtually all living species and some biologically active peptides are long-term used as drugs, peptides have confirmed their good tolerability and safety profiles, providing a credible source of novel drugs for a large number of pathologies such as cancer [39].

In this study, we apply *in silico* protocols to screen FOXM1-targeting peptides and analyze their anti-cancer effects. Using Rosetta suite of computational tools, we first generate a peptide library *in silico* from the FOXM1 N-terminal domain sequence (1-138aa) and predict FOXM1-binding peptide candidates through automated docking protocol. Based on the FOXM1-binding and cell inhibitory results of the candidates, we select the peptide (FOXM1 protein sequence 106-126aa) and then synthesize an interfering peptide named M1-21, which fuses with the TAT cell-penetrating sequence [40] and is produced through the D-retro-inverso peptide strategy. We show that M1-21 binds to FOXM1 and inhibits its transcriptional activities. Furthermore, M1-21 also disrupts the FOXM1:β-catenin interaction and prevents the nuclear importation of β-catenin, thereby inhibiting β-catenin transcriptional activities. M1-21 can significantly inhibit the proliferation and migration of cancer cells in vitro and in vivo without obvious toxic and side effects. Together, M1-21, as an interfering peptide of FOXM1, has potential for the anti-cancer drug development.

## Results

### Screening of TAT-106-126 peptide targeting FOXM1 protein

**Fig. 1:**
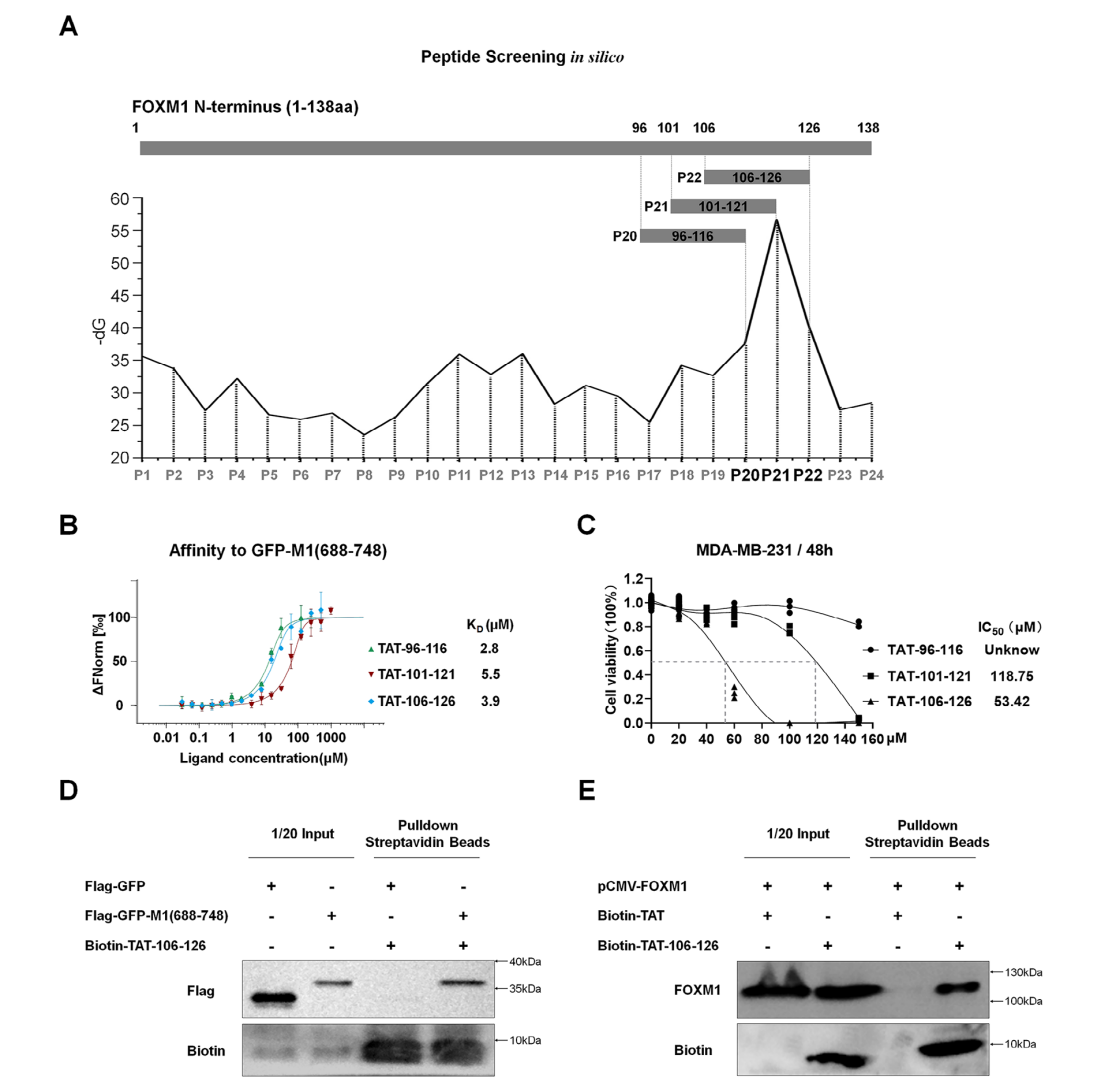
Screening of TAT-106-126 peptide targeting FOXM1 protein. **A** An *in silico* peptide library (P1 to P24, 21-mer) was created from the FOXM1 N-terminus sequence (1-138aa) with a 5 amino acid shift window. The peptide candidates binding to FOXM1 C-terminus (PDB ID 6OSW) were selected by Rosetta FlexPepDock simulation and InterfaceAnalyzer calculation according to docking free energy (dG). **B** P20, P21, and P22 fused with the cell-penetrating TAT sequence (TAT-96-116, TAT-101-121, and TAT-106-126) were produced by solid-phase peptide synthesis. The binding affinity between the synthesized peptides and GFP-FOXM1_688-748_ (GFP-M1(688–748)) was measured by Microscale Thermophoresis (MST) (Monolith NT.115, NanoTemper). Data points indicated the fraction of GFP-M1(688–748)-bound peptides (ΔFNormal/Amplitude) at different concentrations and curves indicated the calculated fits. The error bars represented the standard error of three independent measurements. Mean binding affinity values (K_D_ (µM)) were shown on the panel. Experiments were repeated three times with similar results. **C** MDA-MB-231 cells (2 × 10^5 cells/well) were seeded in 24-well plates for 12 h and treated with different concentrations of TAT-96-116, TAT-101-121, or TAT-106-126 (0, 20, 40, 60, 100, 150 µM). 48 h Later, trypan blue (0.4%) was added to each well for 3 min and the cells were fixed to 4% paraformaldehyde for imaging. The number of viable cells was counted by ImageJ software to calculate the cell viability of each well. The correspondence between cell viability and peptide concentration was plotted by GraphPad software. The mean IC50 value (µM) for each peptide was obtained from three replicates. **D** The TAT-106-126 peptide was biotin-labeled and added to 293T cell lysates (500 µg) expressing exogenous Flag-GFP-FOXM1(688–748) protein. The lysates were incubated with Streptavidin magnetic beads to pull down biotin-peptide/protein complexes. Biotin and Flag-GFP-FOXM1(688–748) protein in samples were detected by Western Blotting. 5% of cell lysates (25 µg) were used as input controls. **E** Biotin-labeled TAT-106-126 or TAT was added to 293T cell lysates (500 µg). The lysates were incubated with Streptavidin magnetic beads to pull down biotin-peptide/protein complexes. Biotin and endogenous FOXM1 protein were detected by Western Blotting. 5% of cell lysates (25 µg) were used as input controls

Our recent study demonstrated that M1-138, a recombinant FOXM1 N-terminal domain (1-138aa) fused with a nine-arginine cell-penetrating peptide, reduced the proliferation and migration of cancer cells [28]. To further screen for FOXM1-targeting interfering peptides, we generated a peptide library *in silico* (P1 to P24, 21-mer) from the sequence of FOXM1 N-terminal domain (1-138aa) with a 5 amino acid shift window. The peptide candidates binding to the FOXM1 C-terminus (PDB ID 6OSW) were selected by Rosetta FlexPepDock simulation [41] and InterfaceAnalyzer calculation [42] according to docking free energy (Fig. 1A). Among them, P20 (96-116aa), P21 (101-121aa), and P22 (106-126aa) were predicted to have a relatively high binding affinity to FOXM1 C-terminus. These three peptides fusing with the cell-penetrating TAT sequence were produced (Fig. S1-S4) and Microscale Thermophoresis confirmed their binding affinity to GFP-FOXM1_688 − 748_ with K_D_ = 2.8 µM for TAT-96-116, K_D_ = 5.5 µM for TAT-101-121, and K_D_ = 3.9 µM for TAT-106-126 (Fig. 1B). Unexpectedly, cell viability assays with breast cancer MDA-MB-231 cells showed that only TAT-106-126, but not TAT-96-116 or TAT-101-121, inhibited the cells obviously with IC50 value around 53.42 µM post 48 h treatment (Fig. 1C). Therefore, we focused on TAT-106-126 and analyzed its binding capacity to FOXM1 protein through Pulldown experiments. Biotin-labeled TAT-106-126 were incubated with cell lysates expressing endogenous FOXM1 protein or exogenous Flag-GFP-FOXM1(688–748) protein and then pulled down with Streptavidin Magnetic Beads, confirming that TAT-106-126 was capable of binding to FOXM1 C-terminus or full-length FOXM1 (Fig. 1D-E). In contrast, although biotin-labeled TAT-96-116 or TAT-101-121 interacted with the FOXM1 C-terminus (Fig. S5A), neither of them effectively bound to full-length FOXM1 in the Pulldown experiments (Fig. S5B), supporting their weak inhibitory effects on cancer cells compared to TAT-106-126.

### M1-21 was more stable than TAT-106-126 and inhibited multiple types of cancer cells

**Fig. 2:**
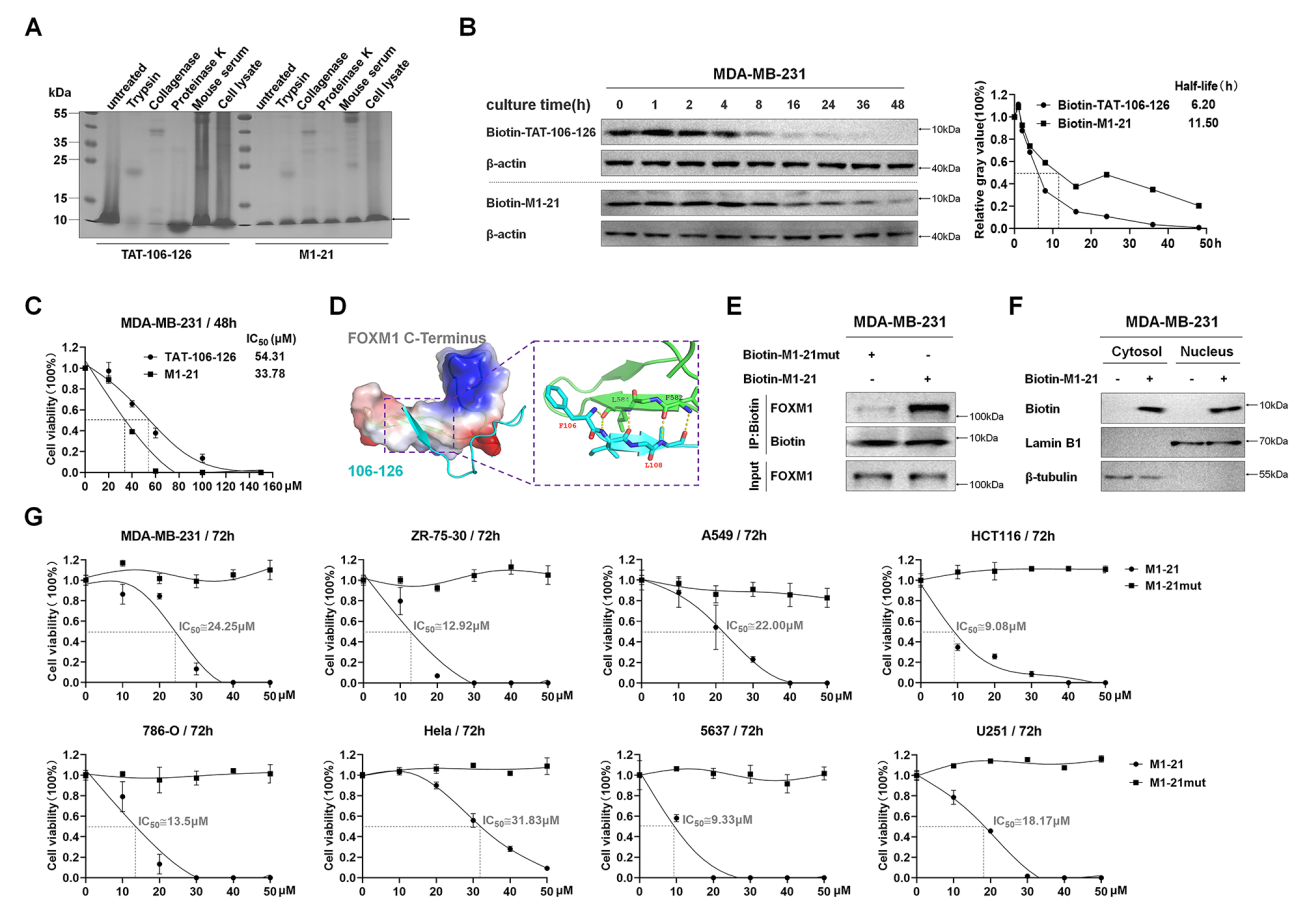
M1-21 was more stable than TAT-106-126 and inhibited multiple types of cancer cells. **A** TAT-106-126 peptide (30 µg) or D-retro-inverso M1-21 peptide (30 µg) was incubated with trypsin (0.1 µg), collagenase (0.1 µg), proteinase K (0.5 µg), mouse serum (20 µg) or cell lysates (20 µg) for 2 h. SDS-PAGE gel electrophoresis and Coomassie brilliant blue staining were performed to measure peptide stability. **B** MDA-MB-231 cells were treated with biotin-labeled TAT-106-126 (20 µM) or biotin-labeled M1-21 (20 µM) for 2 h and then replaced with fresh medium. Cells were collected at different post-treatment time points (0, 1, 2, 4, 8, 16, 24, 36, 48 h). The protein lysates were extracted and detected by Western Blotting. β-actin was used as a loading control. **C** The mean IC50 value (µM) of TAT-106-126 or M1-21 at 48 h to MDA-MB-231 cells was obtained according to the procedure described in Fig. 1C. **D** The docking of peptide P22 (106–126) to the FOXM1 C-terminus (PDB ID 6OSW) was created by Rosetta FlexPepDock and PyMOL. Left, the interface of peptide-protein interaction, the peptide was shown in cyan, and the C-terminus of FOXM1 was shown according to its electrostatic potential. Right, hydrogen bonds are formed by residues at the interface of peptide and protein interaction. **E** Biotin-labeled M1-21 (Biotin-M1-21, 20 µM) or biotin-labeled M1-21mut (Biotin-M1-21mut, 20 µM) was added to MDA-MB-231 cell lysates (500 µg). Streptavidin magnetic beads were added to lysates to pull down the biotin-peptides/protein complexes. Biotin and protein in the samples were detected by Western blotting. 5% of cell lysates (25 µg) were used as input controls. **F** MDA-MB-231 cells were treated with Biotin-M1-21 (20 µM) for 4 h. Cytosol and nuclear protein samples were prepared and detected by Western blotting. β-tubulin or Lamin B1 was used as the loading controls for cytosol or nuclear proteins respectively. **G** The mean IC50 value (µM) of M1-21 at 72 h for multiple cancer cell lines was obtained according to the procedure described in Fig. 1C. M1-21mut was used as the control. Cell lines tested included breast cancer MDA-MB-231 and ZR-75-30, lung adenocarcinoma A549, colon cancer HCT116, renal clear cell tumor 786-O, cervical cancer Hela, bladder cancer 5637, glioma U251

Natural amino acid-composed peptides tended to be degraded by proteases in vivo, and chemical optimization approaches such as D-retro-inverso (DRI) strategy were developed to enhance peptide stability [43]. DRI peptides were composed of D-amino acids assembled in the reverse order of the parent peptides and we synthesized the DRI form of TAT-106-126, M1-21, which theoretically presented an orientation of side chains similar to that of the original peptide (Fig. S6). M1-21 showed much better resistance to degradation than parent TAT-106-126 when incubated with trypsin, collagenase, protease K, mouse serum, or cell lysate (Fig. 2A). Similarly, M1-21 showed a longer half-life (11.5 h) than TAT-106-126 (6.2 h) when the two biotin-labeled peptides were tested in living MDA-MB-231 cells (Fig. 2B).

Consequently, M1-21 showed a stronger inhibitory capacity on MDA-MB-231 cells than TAT-106-126, evidenced by the decreased IC50 value of M1-21 (33.78 µM) compared to TAT-106-126 (54.31 µM) (Fig. 2C). Microscale Thermophoresis analysis using GFP-FOXM1 demonstrated that M1-21 exhibited a comparable FOXM1 binding affinity (K_D_ = 12.5 µM) to that of TAT-106-126 (K_D_ = 7.1 µM) (Fig. S8), indicating that the increased inhibitory effects of M1-21 on cells were due to the enhanced stability of M1-21. We simulated the structure of P22 (106-126aa) binding to FOXM1 C-terminus with Rosetta FlexPepDock and InterfaceAnalyzer, which created high-resolution models between flexible peptides and proteins. F106 and L108 of P22 were predicted to be the key residues to maintain P22’s binding to FOXM1 C-terminus (Fig. 2D) and the synthesized M1-21 mutant (M1-21mut, F106A, and L108A) (Fig. S9) lost the ability to bind to FOXM1 C-terminus or to inhibit cancer cells (Fig. S10). M1-21, but not M1-21mut, bound to endogenous FOXM1 when the two biotin-labeled peptides were incubated with the lysates of MDA-MB-231 cells (Fig. 2E), further confirming that F106 and L108 mediated the interaction between M1-21 and FOXM1. M1-21 could be distributed in cytoplasm and nucleus after entering MDA-MB-231 cells (Fig. 2F). We tested the inhibitory effects of M1-21 on multiple types of cancer cells and found that M1-21 dramatically inhibited all the tested cancer cells, including breast cancer MDA-MB-231 and ZR-75-30, lung adenocarcinoma A549, colon cancer HCT116, renal clear cell tumor 786-O, cervical cancer Hela, bladder cancer 5637, and glioma U251, with variable values of IC50 (Fig. 2G). In addition, we also found that M1-21 did not inhibit MCF-10 A cells, normal breast epithelial cells, at the doses tested (Fig. S11). Thus, we obtained the cell-penetrating DRI peptide M1-21 that could inhibit cancer cells probably by binding to FOXM1.

### The proliferation and migration of MDA-MB-231 cells were inhibited by M1-21

**Fig. 3:**
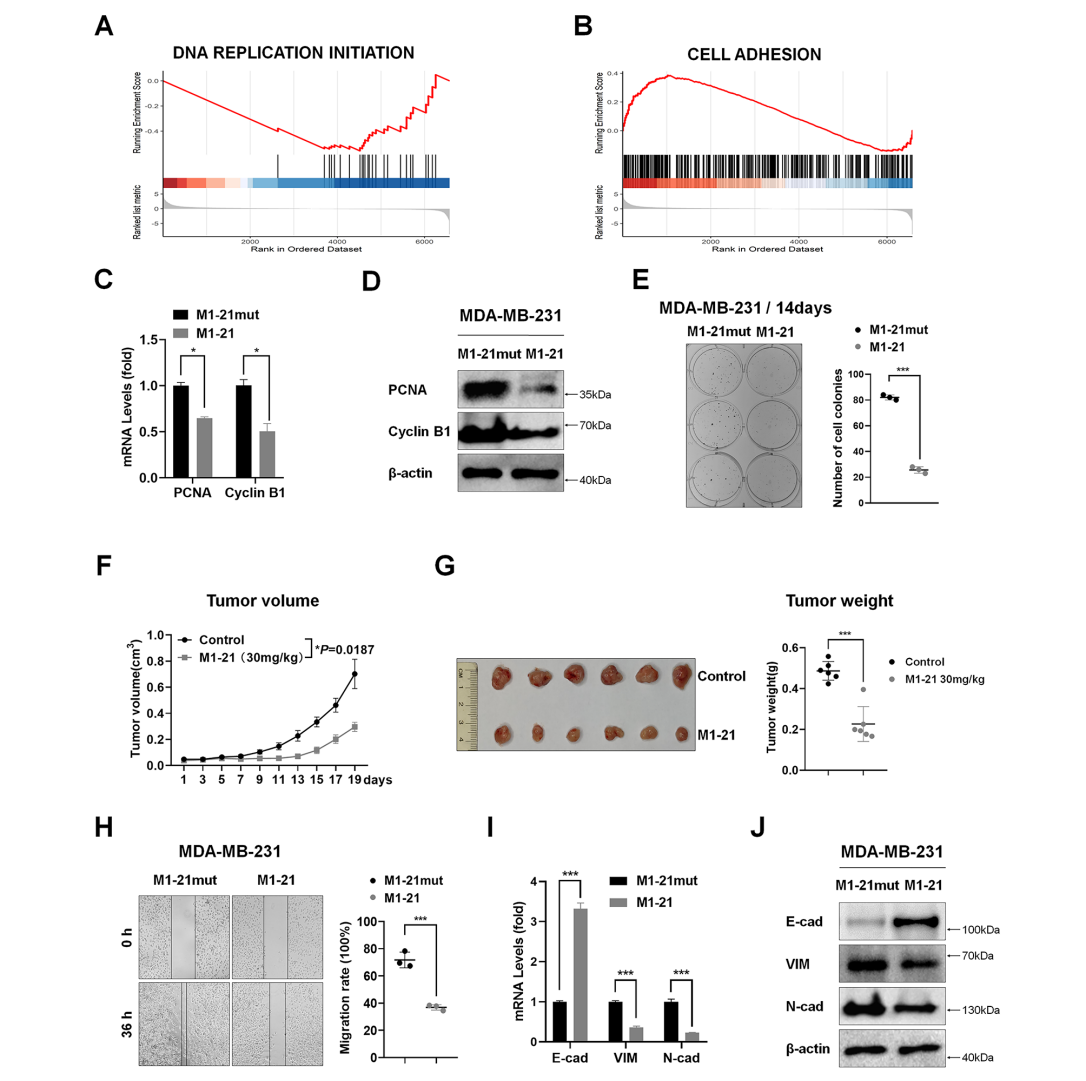
The proliferation and migration of MDA-MB-231 cells were inhibited by M1-21. **A-B** MDA-MB-231 cells were treated with M1-21 or M1-21mut (20 µM) for 24 h and total RNA samples were prepared for RNA sequencing. Gene set enrichment analysis (GSEA) was performed to show inhibition of the genes of DNA replication initiation genes (**A**) and cell adhesion gene activation genes (**B**). **C** qPCR was used to verify changes in PCNA and CyclinB1 mRNA levels from panel A samples. GAPDH was used as loading control. **D** MDA-MB-231 cells were treated as panel A and cell lysates were prepared. PCNA and CyclinB1 protein levels were measured by Western blotting. β-actin was used as a loading control. **E** MDA-MB-231 cells (200 cells/well) were treated with M1-21 or M1-21mut (20 µM) for 14 days. The cells were fixed with 4% paraformaldehyde and stained with crystal violet for imaging. The treatment’s ability to form colonies was measured by counting the number of cell colonies in each well. **F** BALB/c nude mice (female, 6 weeks old) were subcutaneously injected with MDA-MB-231 cells (1 × 10^6 cells/mouse). One week later, the mice were randomly divided into two groups followed by intraperitoneal PBS injection (Control, n = 6) or M1-21 (30 mg/kg, n = 6) once every two days for 19 days. The volume of engrafted tumors was measured every two days and growth curves were obtained at the end of the experiment. The tumor volume (V) was calculated by: V = length × diameter^2^ × 1/2. **G** Images and weight of engrafted tumors on Day 19. **H** MDA-MB-231 cells were seeded into plates to reach 90% confluence. A 200 µl pipette tip was used to thread a line and then photographed. Cells were then treated with M1-21 (10 µM) or M1-21mut (10 µM) and cell migration was recorded at 36 h post-wound formation. Number of repetitions = 3. **I-J** MDA-MB-231 cells were treated with M1-21 (10 µM) or M1-21mut (10 µM) and 24 h later cells were harvested for the preparation of total RNA or cell lysates. The levels of mRNA or protein of E-cadherin (E-cad), Vimentin (VIM), and N-cadherin (N-cad) were measured by qPCR or Western blotting, respectively. GAPDH or β-actin was used as a loading control for mRNA or protein. mRNA values represented the mean ± SD of three replicates, and significance was calculated using unpaired *t* test. *P < 0.05; **P < 0.01; ***P < 0.001

RNA sequencing was performed with mRNA samples from M1-21 or M1-21mut-treated MDA-MB-231 cells. We noticed that the pattern of gene expression in cells exposed to M1-21 treatment (20 µM for 24 h) was dramatically altered by gene set enrichment analysis (GSEA), such as inhibition of the gene set of DNA replication and activation of the gene set of cell adhesion (Fig. 3A-B), suggesting that cell proliferation and migration were affected by M1-21. Consistent with GSEA data, we found that mRNA and protein levels of PCNA and Cyclin B1, selected as proliferation markers, were downregulated by M1-21 in MDA-MB-231 cells (20 µM for 24 h) (Fig. 3C-D). Colony formation assays further confirmed the inhibitory effects of M1-21 on MDA-MB-231 cell proliferation (Fig. 3E). To further confirm that M1-21 inhibited cancer cell proliferation in vivo, we generated mouse tumor-engrafted models by subcutaneously injecting MDA-MB-231 cells into BALB/c nude mice and then treated the mice with intraperitoneal injection of M1-21 (30 mg/kg) once every two days for 3 weeks (Fig. S12A). Treatment with M1-21 resulted in growth inhibition of engrafted tumors (Fig. 3F). Tumors collected at the end of the experiments showed that M1-21 significantly decreased the size and weight of engrafted tumors compared to controls (Fig. 3G), correlated with the decreased levels of FOXM1 and FOXM1-regulated CDC25B and PLK1 in M1-21-treated tumor samples (Fig. S12B). In addition, we found that M1-21 (10 µM for 36 h) inhibited MDA-MB-231 cell migration through Wound Healing assays (Fig. 3H). Consequently, the levels of epithelial marker E-cadherin enhancing cell adhesion were increased and the levels of mesenchymal markers Vimentin and N-cadherin abrogating cell adhesion were decreased by M1-21 treatment in cells (Fig. 3I-J). Thus, we demonstrated that M1-21 inhibited cancer cells by preventing their proliferation and migration.

### M1-21 interacted with multiple regions of FOXM1 protein and inhibited FOXM1-related transcriptional activities

**Fig. 4:**
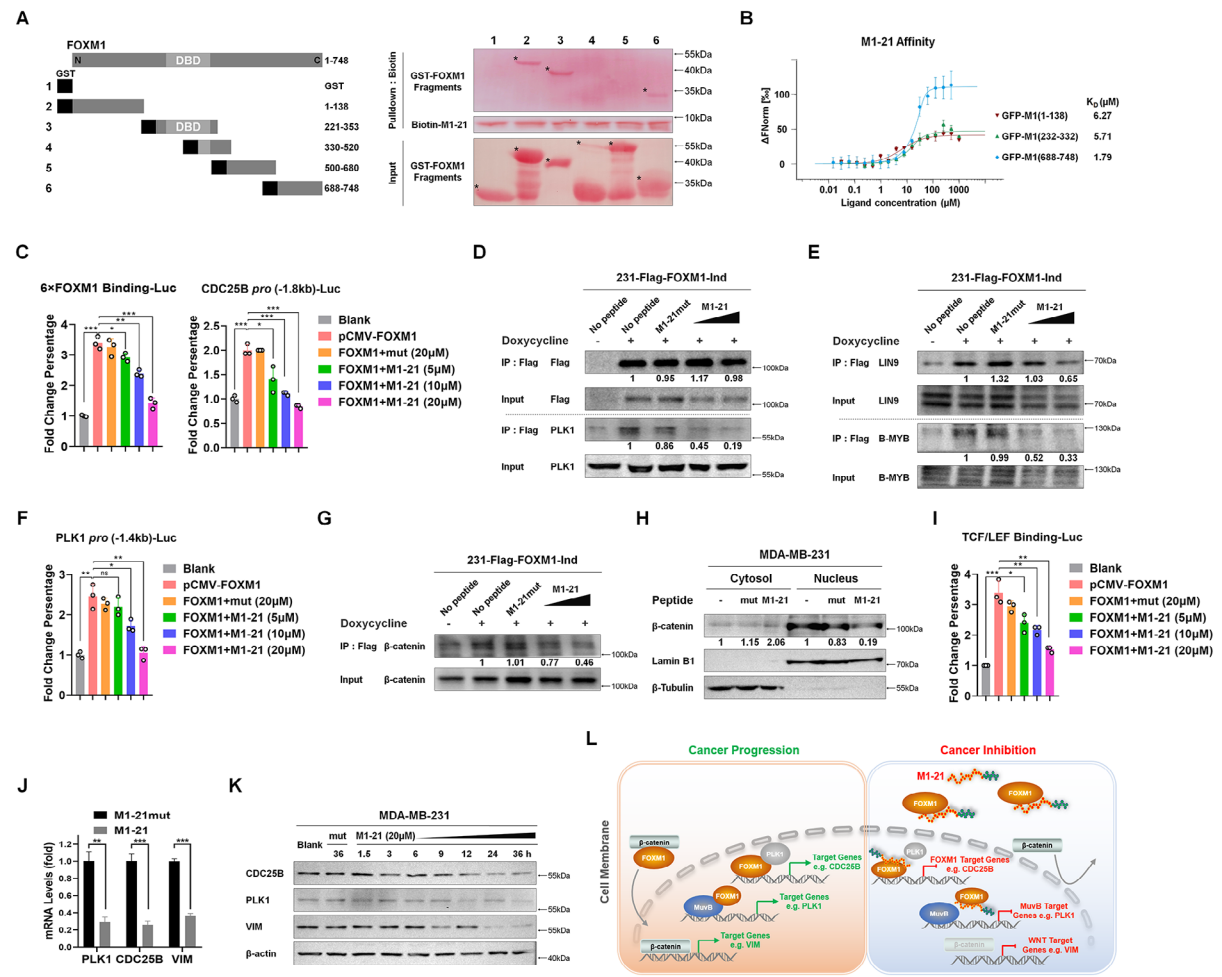
M1-21 interacted with multiple regions of FOXM1 protein and inhibited FOXM1-related transcriptional activities. **A** GST-tagged recombinant proteins fused with different FOXM1 regions (100 µg) (GST-FOXM1_1 − 138_, GST-FOXM1_221 − 353_, GST-FOXM1_330 − 520_, GST-FOXM1_500 − 680_, or GST-FOXM1_688 − 748_) were incubated with Biotin-M1-21 (20 µM) respectively. Streptavidin magnetic beads were added to the mixtures to pull down biotin-M1-21/protein complexes. The recombinant proteins in the samples were separated by PAGE-gel and detected with Ponceau S staining solution (marked by *). 10% of recombinant proteins (10 µg) were used as input controls. **B** The binding affinity of M1-21 to GFP-FOXM1_1 − 138_ (GFP-M1(1-138)), GFP-FOXM1_232 − 332_ (GFP-M1(232–332)), or GFP-FOXM1_688 − 748_ (GFP-M1(688–748)) was measured by MST. Mean binding affinity values (K_D_ (µM)) were shown on the panel. Experiments were repeated three times with similar results. **C** Reporter plasmids containing the luciferase gene downstream of the 6×FOXM1 binding sequence (6×FOXM1 Binding-Luc, 0.4 µg) or the − 1.8 kb CDC25B promoter (CDC25B *pro*(-1.8 kb)-Luc, 0.4 µg) were co-transfected with pCMV-FOXM1 (0.6 µg) into HEK-293T cells, plus pRL-CMV plasmid (20 ng/well) as a loading control. After 24 h, the cells were treated with M1-21mut (20 µM) or M1-21 (5, 10, 20 µM) for another 24 h. Then cell lysates were prepared and used for the measurement of dual Luciferase activities. Number of repetitions = 3. **D** The Flag-FOXM1 inducible MDA-MB-231 cell line (231-Flag-FOXM1-Ind) was induced with doxycycline (200 ng/mL) for 24 h and then treated with M1-21mut (20 µM) or M1-21 (10, 20 µM) for 6 h. Cell lysates (500 µg) were extracted and incubated with anti-Flag magnetic beads to pull down Flag-FOXM1/proteins complexes. The levels of Flag-FOXM1 and PLK1 proteins in the samples were detected by Western blotting. 5% of cell lysates (25 µg) were used as input controls. **E** Flag-FOXM1, LIN9, and B-MYB protein levels in panel D samples were detected by Western blotting. **F** Reporter plasmids containing the luciferase gene downstream of the − 1.4 kb PLK1 promoter (PLK1 *pro*(-1.4 kb)-Luc, 0.4 µg) were co-transfected with pCMV-FOXM1 (0.6 µg) into HEK-293T cells, plus pRL-CMV plasmid (20 ng/well) as loading control. After 24 h, cells were treated with M1-21mut (20 µM) or M1-21 (5, 10, 20 µM) for another 24 h. Then cell lysates were prepared and used to measure dual Luciferase activity. Number of repetitions = 3. **G** Flag-FOXM1 and β-catenin levels in panel D samples were detected by Western blotting. **H** MDA-MB-231 cells were treated with M1-21mut (20 µM) or M1-21 (20 µM) for 6 h. Cytosol and nuclear protein samples were prepared and β-catenin levels were detected by Western blotting. β-tubulin or Lamin B1 was used as the loading controls for cytosol or nuclear proteins respectively. **I** TCF/LEF Binging-Luc reporter plasmid (0.4 µg) was co-transfected with pCMV-FOXM1 (0.6 µg) into HEK-293T cells, plus pRL-CMV plasmid (20 ng/well) as loading control. After 24 h, cells were treated with M1-21mut (20 µM) or M1-21 (5, 10, 20 µM) for another 24 h. Then cell lysates were prepared and used to measure dual Luciferase activity. Number of repetitions = 3. **J** MDA-MB-231 cells were treated with M1-21mut (20 µM) or M1-21 (20 µM) for 24 h. The mRNA levels of PLK1, CDC25B, and Vimentin (VIM) were measured by qPCR. GAPDH was used as a loading control. **K** MDA-MB-231 cells were treated with M1-21mut (20 µM) or M1-21 (20 µM) at different time points (1.5, 3, 6, 9, 12, 24, 36 h). The protein levels of PLK1, CDC25B, and Vimentin (VIM) were measured by Western blotting. β-actin was used as a loading control. **L** The diagram depicts the molecular mechanisms of M1-21 inhibiting FOXM1-related transcriptional activities. mRNA values represented the mean ± SD of three replicates, and significance was calculated using unpaired *t* test. *P < 0.05, **P < 0.01, ***P < 0.001

M1-21 was selected and synthesized to bind FOXM1 in cancer cells. To further investigate how M1-21 interfered with FOXM1-related transcriptional activities, we generated multiple truncated FOXM1 recombinant proteins tagged by GST and identified FOXM1 regions in FOXM1 bound by biotin-labeled M1-21 via Pulldown assays. We noticed that M1-21 not only bound to GST-FOXM1_688 − 748_ as expected, but also GST-FOXM1_1 − 138_ or GST-FOXM1_221 − 353_ surprisingly (Fig. 4A). Microscale Thermophoresis (MST) was performed to confirm the binding of M1-21 to these different FOXM1 domains at relatively high affinity (K_D_ = 6.27 µM to GFP-FOXM1_1 − 138_, K_D_ = 5.71 µM to GFP-FOXM1_232 − 332_, and K_D_ = 1.79 µM to GFP-FOXM1_688 − 748_) (Fig. 4B), while M1-21mut lost its binding ability to all these three domains of FOXM1 (Fig. S10B and Fig. S13). The three domains contributed to FOXM1-related transcriptional activities (FOXM1_1 − 138_ for interaction with the MuvB complex [8, 9], FOXM1_232 − 332_ for DNA binding [7], FOXM1_688 − 748_ for interaction with the CBP complex [11, 12]) on the expression of its downstream genes. First, we found that M1-21 did not disturb the DNA binding ability of FOXM1 to its putative binding sequence in Electrophoretic Mobility Shift Assays (EMSAs) (Fig. S14A) and docking simulation predicted that P22 (M1-21 parent peptide) interacted with S251 and D268 of FOXM1 DBD (Fig. S14B), both residues not mediating FOXM1-DNA binding. Second, to analyze M1-21 binding to FOXM1_688 − 748_ to disturb the direct transcriptional activation by FOXM1, we confirmed that M1-21 abrogated FOXM1-mediated stimulation on FOXM1-binding promoters (an artificial 6xFOXM1 binding sequence-containing promoter or an endogenous − 1.8 kb promoter of CDC25B) in cotransfection experiments (Fig. 4C). The PLK1 protein kinase interacted with FOXM1 C-terminus and phosphorylated certain residues such as Ser702, Ser715 or Ser724 in this domain, which then recruited the CBP complex to activate downstream genes [12]. Using a home-made MDA-MB-231 cell line that could be induced to express Flag-FOXM1 (231-Flag-FOXM1-Ind) (Fig. S15), we verified that M1-21 disrupted the interaction between FOXM1 and PLK1 by Co-IP experiments (Fig. 4D), explaining that M1-21 suppressed FOXM1-mediated direct transcriptional activation. Additionally, we discovered that the parent peptide of M1-21, TAT-106-126, exhibited comparable activity to M1-21 in disrupting the interaction between FOXM1 and PLK1 (Fig S16). Third, to analyze M1-21 binding to FOXM1_1 − 138_ to disrupt indirect transcriptional activation by the FOXM1-MuvB complex on the CHR motif, we performed Co-IP experiments to confirm that M1-21 disrupted the interaction of FOXM1:LIN9 or FOXM1: B-MYB in a dose-dependent manner (Fig. 4E). Due to that both LIN9 and B-MYB were required to recruit FOXM1 to the MuvB complex [8, 9], these results explained how M1-21 repressed FOXM1-MuvB-dependent gene activation. This idea was further supported by the results of cotransfection experiments, in which M1-21 abolished FOXM1-MuvB-mediated stimulation on the CHR-containing promoter (an endogenous − 1.4 kb promoter of PLK1, containing CHR sites at -30 to -25, -89 to -84, -149 to -144 bp but no perfect FKH site) (Fig. 4F).

Because FOXM1 mediated the nucleus-translocation of β-catenin for promoting the activation of the WNT signaling pathway and FOXM1 DBD was required for the interaction between FOXM1 and β-catenin [13], we intended to ask whether M1-21 interfered with the functions of β-catenin by binding to FOXM1_232 − 332_. GSEA analysis showed that the gene set of the WNT signaling pathway was inhibited by M1-21 in MDA-MB-231 cells (Fig. S17), implicating that M1-21 repressed the activities of β-catenin. The Co-IP experiments showed that M1-21 disrupted the interaction between FOXM1 and β-catenin (Fig. 4G). Consequently, M1-21 resulted in elevated levels of β-catenin in the cytoplasm and declined levels of β-catenin in the nucleus (Fig. 4H). In addition, M1-21 abolished β-catenin-mediated stimulation on the TCF/LEF-binding promoter (Fig. 4I). Finally, we measured the levels of mRNA and protein of three typical genes (CDC25B as the FOXM1 direct target gene [44], PLK1 as the FOXM1 indirect target gene [8], and Vimentin as the β-catenin target gene [45]) post M1-21 treatment in MDA-MB-231 cells and found that M1-21 dramatically repressed the expression of all of them (Fig. 4J-K), further supporting the molecular mechanisms of M1-21 inhibiting the FOXM1-related transcriptional activities summarized in a diagram (Fig. 4L).

### M1-21 inhibited cancer proliferation and metastasis in wild-type mice

**Fig. 5:**
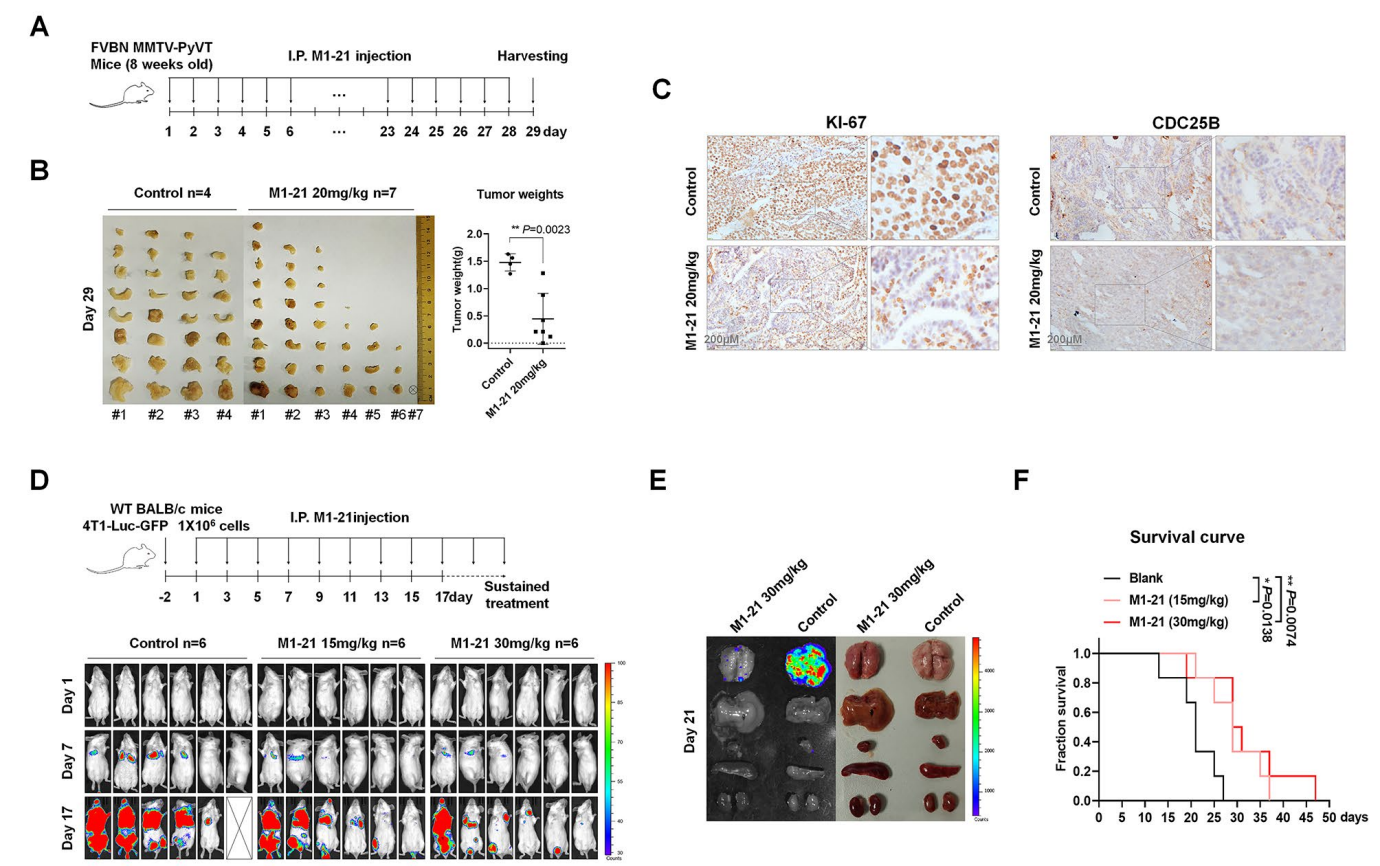
M1-21 inhibited cancer proliferation and metastasis in wild-type mice. **A** The FVB/N MMTV-PyVT mice (female, 8 weeks old, beginning to form spontaneous breast cancer) were injected intraperitoneally with PBS (Control, n = 4) or M1-21 (20 mg/kg, n = 7) once daily for 28 days. **B** Cancer tissue was harvested on Day 29 for imaging. The weight of cancer tissue was measured for GraphPad analysis. **C** The representative sections of the cancer samples of panel A were immunostained with anti-KI-67 antibody (1:200), and anti-CDC25B antibody (1:200) followed by microscope imaging (200×, Nikon TE2000). Scale bar: 200 μm. **D** Wild-type BALB/c mice were tail-vein injected with 4T1-Luc-GFP cells (1 × 10^6 cells/mouse). Three days later, the mice were randomly divided into three groups, followed by intraperitoneal PBS injection (Control, n = 6), M1-21 (15 mg/kg, n = 6), or M1-21 (30 mg/kg, n = 6) every two days throughout the experiment. In vivo imaging of mice was performed by intraperitoneal injection of D-Luciferin potassium salt (3 mg/200 mL/mouse) at various post-treatment time points M1-21 (Day 1, Day 7, and Day 17) and then photographs were taken by the IVIS Lumina XR machine. Bright red fluorescence signals represented the amount of luciferase-labeled 4T1 cells in mice. **E** Different organs of representative mice were harvested on Day 21 and fluorescent imaging was performed as described above. **F** The D panel mouse survival statistic curves were obtained by the Mantel-Cox estimator with log rank test

Because we intended to analyze the anti-cancer effects of M1-21 in mice with wild-type backgrounds, we had to make sure that M1-21 could bind to mouse Foxm1. The three domains of human FOXM1 (1-138aa, 221-353aa, and 688-748aa) were highly conserved with that of mouse Foxm1 (Fig. S18A). Biotin-labeled M1-21 could bind to endogenous Foxm1 when incubated with the lysates of mouse breast cancer 4T1 cells (Fig. S18B) and as expected, M1-21 inhibited mouse 4T1 cells at a similar concentration (20 µM) inhibiting human MDA-MB-231 cells (Fig. S18C), suggesting that M1-21 could inhibit mouse cancers by targeting mouse Foxm1. Then, we used FVBN MMTV-PyVT mice, which possessed intact immune systems and developed spontaneous breast cancers around Week 8 after birth [46], to test the anti-cancer effects of M1-21. FVBN MMTV-PyVT mice (female, 8 weeks old) were injected intraperitoneally with PBS (Control, n = 4) or M1-21 (20 mg/kg, n = 7) one time a day for 28 days. The mice were photographed every week afterward for the observation of cancer progression (Fig. S19) and cancer tissue from the animals was harvested on Day 29. We noticed that the M1-21 treatment dramatically decreased the number of formed cancers in animals. Of the seven M1-21-treated mice, six mice had obvious therapeutic benefits and even one mouse was cancer-free at the end of the experiment (Fig. 5B). The weight of total cancer tissue from each mouse was measured to show that M1-21 inhibited cancer progression in the animals (Fig. 5B). Representative immunostaining from cancer sample sections confirmed that the levels of KI-67, a cell proliferation marker, and CDC25B, the FOXM1 downstream target gene, were dramatically down-regulated (Fig. 5C). Thus, these results confirmed that M1-21 inhibited the proliferation of cancers in wild-type mice.

To test the inhibitory effects of M1-21 on the metastasis of cancer cells in mice with wild-type background, we constructed a mouse-stable luciferase-GFP-expressed cell line with 4T1 cell line (4T1-Luc-GFP) that allowed in vivo cancer cell tracking (Fig. S20). Due to 4T1 cells possessing BALB/c mouse background, we injected 4T1-Luc-GFP cells (1 × 10^6 cells/mouse) into BALB/c mice (female, n = 18) via tail-vein. Three days later, the mice were randomly divided into three groups, followed by intraperitoneal injection of PBS (Control, n = 6), M1-21 (15 mg/kg, n = 6), or M1-21 (30 mg/kg, n = 6) once every two days until the end of the experiments. At different time points post the M1-21 treatment (Day 1, Day 7, and Day 17), the in vivo imaging of the mice was performed by the intraperitoneal injection of D-Luciferin potassium salt (3 mg/200 µL/mouse). The luminaire photos showed that M1-21 prevented metastasis of 4T1-Luc-GFP cells in animals (Fig. 5D). Different organs of representative mice were harvested on Day 21, and luminaire imaging showed that M1-21 prevented metastasis of 4T1-Luc-GFP cells mainly to lung of the animals (Fig. 5E). The survival of the animals during the experiments was monitored and the survival statistic curve showed that M1-21 significantly prolonged the survival time of the animals (Fig. 5F).

### Distribution and toxicity analysis of M1-21 in wild-type mice

**Fig. 6:**
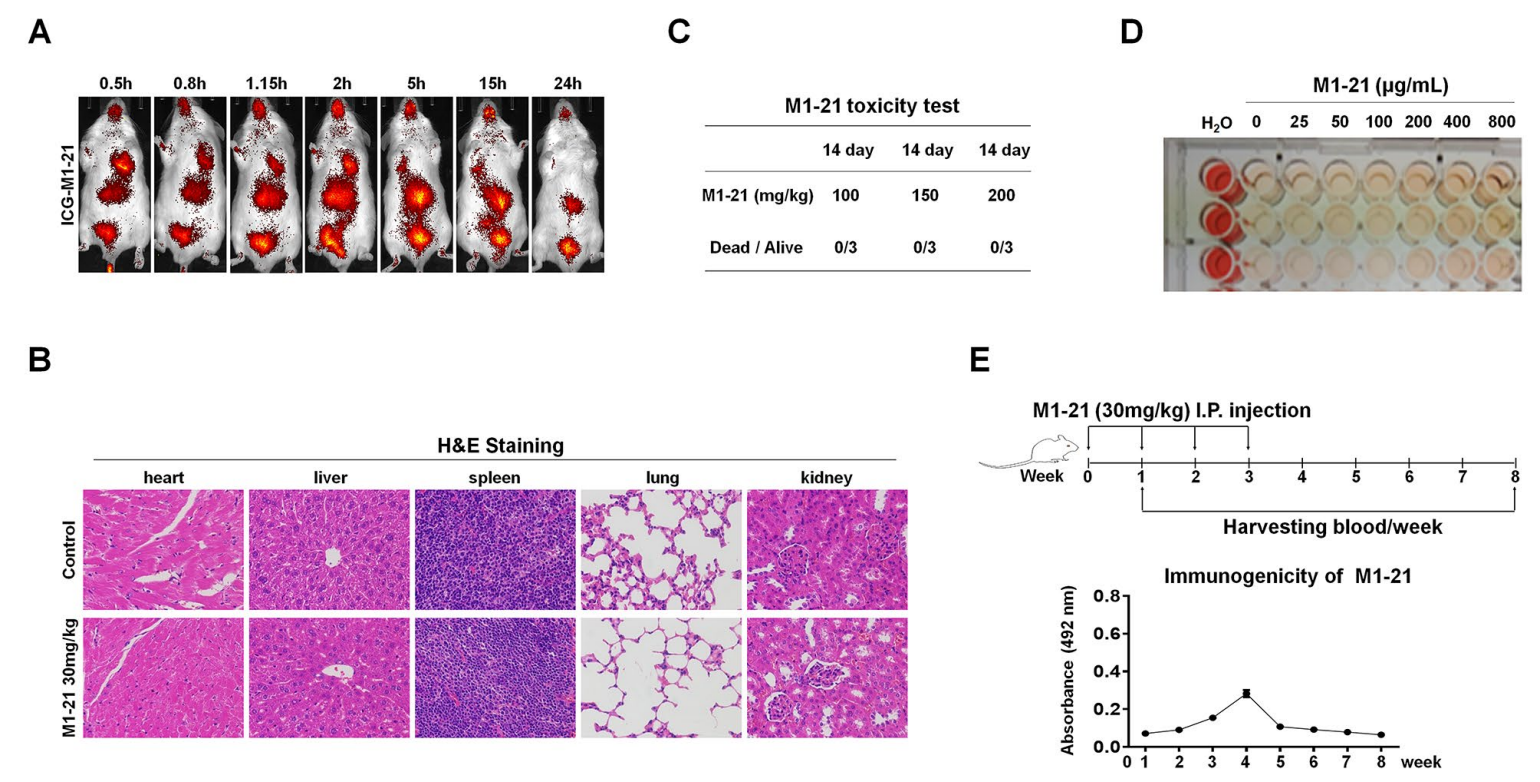
Distribution and toxicity analysis of M1-21 in wild-type mice. **A** ICG-labeled M1-21 (ICG-M1-21) (30 mg/kg) was tail-vein injected into wild-type ICR/JCL mice (female, 6 weeks old). Imaging animals at different post-treatment time points (0.5, 0.8, 1.15, 2, 5, 15, 24 h) was performed with the IVIS Lumina XR machine under 785 nm excitation light. **B** WT ICR/JCL mice (female, 6 weeks old) were intraperitoneally injected with PBS (Control) or M1-21 (30 mg/kg) every two days. One week later, the animals’ different organs were harvested and subjected to tissue sectioning and H&E staining. Photos were taken by inverted microscope (400×, Nikon TE2000). **C** Acute toxicity of M1-21 was measured by a single intraperitoneal injection of M1-21 (100 mg/kg, 150 mg/kg, or 200 mg/kg) in ICR/JCL mice (female, 6 weeks old, n = 3 for each group). The mice’s diet and activity were observed continuously for 14 days. **D** M1-21 hemolysis was measured by incubating mouse blood with M1-21 at different concentrations (25, 50, 100, 200, 400, 800 µg/mL). Water and PBS were used as hemolysis positive and negative controls, respectively. The absorbance of the samples was measured at 540 nm and used to calculate the Hemolysis rate % = [(OD Sample - OD Negative) / (OD Positive - OD Negative)] × 100%. **E** Immunogenicity analysis of M1-21. ICR/JCL mice (female, 6 weeks old) were injected intraperitoneally with M1-21 (30 mg/kg) once a week for 4 weeks. Blood samples were collected from mice at different time points (Week 2 to Week 8) and the absorption of anti-M1-21 antibody in serum (1:1000 dilution) was measured by ELISA. HRP-conjugated anti-mouse IgG was used as an ELISA secondary antibody. Absorption was measured at 492 nm

To explore the distribution of M1-21 in mice, we injected ICG-labeled M1-21 (ICG-M1-21, 30 mg/kg) into wild-type ICR/JCL mice via tail vein for in vivo imaging at different post-treatment time points. We found that M1-21 could distribute broadly in the animal and stay in the body for a reasonably long period (> 15 h) (Fig. 6A), implicating that M1-21 possessed good stability in vivo. H&E staining on tissue sections from M1-21-injected ICR/JCL mice (30 mg/kg, once every two days for three weeks) showed no obvious morphological lesions in multiple organs such as heart, liver, spleen, lung, and kidney (Fig. 6B), suggesting that multiple injections of M1-21 at the therapeutic dosage did not cause damage to the vital organs of the animals. We also found that wild-type ICR/JCL mice could tolerate the dosage of M1-21 as high as 200 mg/kg body weight by intraperitoneal (I.P.) injection with no observed toxicity (Fig. 6C). From the hemolytic test of M1-21 to blood cells, we found that a relatively high concentration of M1-21 treatment (800 µg/mL) resulted in very mild hemolysis to erythrocytes (Fig. 6D), proving that M1-21 was well tolerated by normal cells. To test the potential immunogenicity of M1-21 in vivo, we treated wild-type ICR/JCL mice with M1-21 (30 mg/kg/week×4 weeks, I.P. injection) and collected the serum of the animals at different time points (Week 2 to Week 8). ELISA assays were performed to detect anti-M1-21 antibodies in serum, which showed relatively low levels of M1-21-specific antibody generation at the time points tested except at the Week 4-time point (Fig. 6E), indicating an acceptable immunogenicity of M1-21 in vivo. Together, these results showed that M1-21 was well tolerated and safe at its dosage of anti-cancer therapy in vivo.

## Discussion

Although breast cancer in vitro and in vivo models were mainly used for analyzing the anti-cancer effects of M1-21 in this study, we believe that M1-21 possesses a broad spectrum inhibiting multiple types of cancer because of its targeting to FOXM1. As one of the key transcription factors promoting cell proliferation and metastasis, FOXM1 is over-expressed in almost all the types of clinical cancers from the TCGA database [3, 47] and the knockout of FOXM1 in multiple mouse organs inhibits the cancer development of corresponding organs, such as liver [48], lung [49], rectum [50], and ovarian cancer [51]. Interestingly, in addition to the fact that FOXM1-mediated gene expression was suppressed by M1-21, we observed that M1-21 could directly decrease the levels (mRNA or protein) of FOXM1 itself in the cancer cells tested or in spontaneously developed cancer tissues (Fig. S21), probably due to the self-stimulating loop existing in FOXM1-regulated transcription mechanisms of cells [3, 52]. Based on the fact that M1-21 inhibited both FOXM1 expression and function, we observed that M1-21 inhibited a range of cancer types in addition to breast cancer, such as lung adenocarcinoma, colon cancer, renal clear cell tumor, cervical cancer, bladder cancer, and glioma U251, in cell culture (see above). The anti-cancer effects of M1-21 on various solid tumors in vivo need further investigation. Moreover, FOXM1 has been implicated in the pathogenesis of diverse non-neoplastic disorders including pulmonary fibrosis [53, 54] and Maturity-Onset Diabetes [55]. Further research is therefore warranted to evaluate the impact of M1-21 in non-neoplastic conditions.

In this study, we initiated with the peptide P22 which was predicted to bind FOXM1 C-terminus using an *in silico* automated docking approach. However, we observed that M1-21, a derived peptide from P22, not only bound to FOXM1 C-terminus but also interacted with FOXM1 N-terminus (1-138aa) and FOXM1 DBD (221-353aa). This indicates that M1-21 has the potential to interact with multiple domains of the target protein or even other proteins, which could be an advantage in clinical applications. Targeting single domains of proteins with inhibitors often leads to drug resistance, thereby potentially limiting therapeutic durability [56]. We confirmed that M1-21 inhibited the functions of both FOXM1 and β-catenin, thereby inhibiting cancer cells across multiple aspects of phenotypes. We are currently performing mass spectrometry analysis on M1-21 pull-down lysate samples to identify proteins that interact with M1-21. This will give us a better understanding of M1-21’s impact on cancer-related pathways. We have already found CDK1 and XPO1 on the list of M1-21-interacting candidates with high confidence (data not shown). CDK1 is a key protein kinase in the G2/M phase and stimulates cell cycle progression [57]. Therefore, it has been considered a therapeutic target in multiple cancers [58]. XPO1 plays a role in drug resistance [59], and its inhibitor is currently on the market for cancer treatment [60]. It would be worthwhile testing whether M1-21 acts as an inhibitor for CDK1 or XPO1 in future studies, as it might inhibit cancers synergistically by targeting multiple oncoproteins at the same time. In theory, this would also provide evidence that M1-21 outperforms FOXM1 small molecule inhibitors in cancer therapy.

M1-21 is a cell-penetrating peptide that was developed by combining the TAT cell-penetrating sequence. TAT peptide, which is derived from HIV, is used to transport various cargos into cells due to its high solubility and wide range of targets [40]. To date, more than 100 peptide sequences have been identified that are capable of penetrating the plasma membrane [61]. It is generally accepted that penetrating peptides enter cells via an energy-dependent endocytosis or by physical endocytosis through direct cell membrane translocation [62]. However, since TAT does not have cell selection ability, M1-21 is limited in its ability to specifically target cancer cells. To overcome this, cell type-specific penetrating peptides that can enter only certain types of cancer cells are available and can be utilized to deliver bioactive substances into specific cancer cells, without affecting normal or other types of cancer cells [63]. Therefore, it is worth studying in the future whether modifying M1-21 with cell type-specific penetrating peptides can improve its targeting and reduce its cytotoxicity for the treatment of specific cancer types.

This study aimed to investigate the effectiveness of M1-21 in vivo against cancer by utilizing three distinct mouse models. Firstly, a subcutaneous tumor-grafted BALB/c nude mouse model was employed to showcase M1-21’s potential for repressing cancer cell proliferation. To better replicate real-world cancer therapy, it is necessary to use an animal model with an intact immune system to test the efficacy of drugs [64]. Furthermore, due to the effect of the tumor microenvironment on drug action, a model with spontaneous cancers is desirable [65]. The second model, FVBN MMTV-PyVT mice, had spontaneous breast cancers with an intact immune system and was used to showcase the anti-proliferation effects of M1-21 under in vivo conditions similar to real-life scenarios. Finally, we used BALB/c wild-type mice as the third model, along with BALB/c 4T1 mouse breast cancer cells to demonstrate how M1-21 could prevent metastasis in vivo. These in vivo studies present promising pharmacological data that can serve as the foundation for future clinical trials of M1-21. Gaining necessary insight into the effectiveness of M1-21 in different mouse models is crucial in the development of this drug for clinical use, and our results pave the way for future advancements in cancer therapy using M1-21.

## Conclusions

In this study, we utilized *in silico* methodologies to screen peptides targeting FOXM1 and synthesized M1-21, which is a DRI peptide derived from the selected original peptide. With significantly improved characteristics in terms of stability and cell inhibitory activity as compared to the parent peptide, M1-21 displayed high affinity for multiple regions of FOXM1 and effectively interfered with the protein-protein interactions between FOXM1 and its various known partner proteins. Consequently, M1-21 was able to repress FOXM1-related transcriptional activities. Notably, our findings indicate that M1-21 displays promising potential as an anti-cancer agent due to its ability to inhibit the proliferation and migration of cancer cells both in vitro and in vivo without any observable toxic or side effects.

## Materials and methods

### The complete materials and methods were described in supplementary materials

#### Interfering peptide screening by molecular docking simulation

An *in silico* peptide library (P1 to P24, 21-mer) was created based on the FOXM1 N-terminus sequence (1-138aa) with a 5 amino acid shift window. The FOXM1 C-terminus (PDB ID 6OSW) 3D structure was obtained from the RCSB PDB database. Rosetta suite FlexPepDocking [41] was used for peptide-protein docking. The top 1 conformation from 50,000 docking simulations for each peptide was selected based on the docking free energy (dG) calculation with Rosetta InterfaceAnalyzer [42] and PyMOL was used to display the binding interface between peptides and proteins. The negative value of the selected dG was used to represent the binding affinity between each peptide and the target protein.

### Solid-phase synthesis of peptides

Peptide synthesis was performed by solid phase synthesis according to the machine manufacturer’s manual (CS136, CS BIO, USA). The synthesized peptides were purified by reverse phase chromatography (AKTA Purifier, GE, USA). The purified peptides were freeze-dried at -40 °C, stored at -80 °C, and re-dissolved in 1×PBS (0.01 M). The molecular weight of the peptides was confirmed by Matrix-Assisted Laser Desorption/Ionization Time of Flight Mass Spectrometry (UltrafleXtreme, BURKER, Germany) and the purity of peptides was determined by High Performance Liquid Chromatography (LC-2010, RAINBOW, China).

### Microscale thermophoresis assays

Fluorescently labeled proteins were mixed with varying concentrations of chemically synthesized peptides (ranging from 0.030 to 1000 µM) in PBST buffer containing 1×PBS (0.01 M) and 0.05% Tween-20. Approximately 4–6 µL of each sample was loaded in a fused silica capillary (NanoTemper, Germany). Measurements were performed in a Monolith NT.115 (NanoTemper) instrument (set 25℃) at a constant LED power of 60% and MST power of the medium. The data were then analyzed by MO. Affinity Analysis v2.3 NT software (NanoTemper) to determine interaction parameters. Signal-to-noise ratios above 10 were considered significant as suggested by NanoTemper. Data point binding curves from three independent MST measurements are shown, indicating the fraction of peptides-bound GFP-proteins (ΔFNormal/Amplitude) at different ligand concentrations and curves indicate the calculated fits. Error bars represent the Standard Error of three independent measurements.

### Cell lines and cell culture

ZR-75-30, MDA-MB-231, HCT116, 786-O, 5637, A549, Hela, and HEK 293T cells were obtained from ATCC (Manassas, USA). U251 and MCF-10 A were obtained from Procell (Wuhan, China). The 4T1 cell line was obtained from Jiang’s lab (Hunan University, Changsha, China). ZR-75-30, HCT116, 786-O, and 5637 cells were cultured with RPMI-1640 (Sangon Biotech, China). MDA-MB-231, HEK-293T, Hela, U251, A549, and 4T1 cells were cultured with DMEM high glucose medium (Gibco, USA). Culture media were added 10% fetal bovine serum (Gibco) and 1% Penicillin-streptomycin-gentamicin solution. MCF-10 A cells were cultured in DMEM/F12+5% HS+20ng/mL EGF+0.5 µg/mL Hydrocortisone+10 µg/mL Insulin+1% NEAA medium (Procell, China). All cells were cultured in a 5% CO2-humidified atmosphere at 37 °C.

### Preparation of lentivirus and construction of cell lines

pLVX-TetOne-Flag-FOXM1b or pLVX-EF1α-Luc-IRES-EGFP plasmids plus lentiviral packaging plasmids psPAX2 (Addgene #12,259, USA), and pMD2.G (Addgene #12,259, USA) were transfected into HEK-293T cells with a cell confluence of 80% with polyethyleneimine (PEI) transfection reagent (pLVX/psPAX2/pMD2G = 12 µg/9 µg/6 µg). After 8 h, the medium was replaced with fresh medium, and two batches of virus supernatant were respectively harvested at 48 and 72 h. The virus particles were concentrated by running a cryogenic ultracentrifuge at 25,000 RPM for 2 h and redissolved in cold PBS.

Cells (2 × 10^5 cells/dish) in good condition were infected with lentivirus and the fresh medium was changed after 24 h. After additional culture for 48 h, puromycin (1 µg/mL) was added to screen positive cells for one week. Monoclonal cells were collected and cultured and part of the cells were collected to extract the protein for Western blotting to confirm the expression of the target protein.

### RNA-seq analysis

Total RNA was extracted with an RNeasy mini kit (QIAGEN, China). RNA sequencing was performed by Majorbio Co., Ltd. (Shanghai, China). Gene expression levels were finally quantified as Fragments Per Kilobase exon model per million mapped fragments (FPKM). The DEGseq R-pack [66] was used to analyze the differential expression of genes, and genes with |log2(fold change)| > 1 and p.adjust < 0.1 were considered differentially expressed. Genes with p-value < 0.1 between the M1-21 and M21mut groups were used to perform gene set enrichment analysis (GSEA) via the R package ClusterProfiler [67]. The pathways with the normalized enrichment score |NES| > 1 and p-value < 0.05 were considered to be significantly enriched.

### Anti-cancer effects of M1-21 in vivo

BALB/c nude mice and BALB/c wild-type mice (female, 6 weeks old) were purchased from Hunan SJA Laboratory Animal Co., Ltd. (Changsha, China). FVB/N-Tg(MMTV-PyVT)634Mul/J transgenic mice [46] were purchased from The Jackson Laboratory (Bar Harbor, ME, USA). BALB/c nude mice were injected subcutaneously with MDA-MB-231 cells (1 × 10^6 cell/mouse). 7 days later, the mice were randomly divided into two groups (Control n = 6 and M1-21 n = 6) and M1-21 treatment was initiated. Tumor size was measured every two days. The tumor volume (V) was calculated by: V = length × diameter^2^ × 1/2. Cancer tissue from the animals was harvested for imaging and weighing on Day 19. FVB/N-Tg (MMTV-PyVT) mice (female, 8 weeks old) were injected intraperitoneally with PBS (Control, n = 4) or M1-21 (20 mg/kg, n = 7) once daily for 28 days. The mice were photographed every week thereafter. Cancer tissue from the animals was harvested on Day 29. The weight of the total cancer tissue from each mouse was measured and immunostaining sections of the cancer sample were performed for selected proteins. Wild-type BALB/c mice were injected with 4T1-Luc-GFP cells (1 × 10^6 cells/mouse) via the tail vein. 3 days later, the mice were randomly divided into three groups, followed by intraperitoneal PBS injection (Control, n = 6), M1-21 (15 mg/kg, n = 6), or M1-21 (30 mg/kg, n = 6) every two days until the end of the experiments. In vivo imaging of mice was performed by intraperitoneal injection of D-Luciferin potassium salt (3 mg/200 mL/mouse) with IVIS Lumina XR machine (Caliper, USA) at different time points after M1-21 treatment (Day 1, Day 7, and Day 17). Different organs of representative mice were harvested on Day 21 for luminaire imaging. When the mice lost mobility and had difficulty eating, the mice were considered near death and terminated according to animal welfare. The survival statistic curve was obtained by the Mantel-Cox estimator with the log rank test.

### Distribution and toxicity analysis of M1-21

Wild type ICR/JCL mice (6 weeks old) were purchased from Hunan SJA Laboratory Animal Co., Ltd. (Changsha, China). ICR/JCL mice were injected with ICG (indocyanine green) labeled M1-21 (ICG-M1-21, 30 mg/kg) via tail vein for in vivo imaging (IVIS Lumina XR, Caliper, USA) at different time points to explore the distribution of M1-21 in mice. ICR/JCL mice were injected with M1-21 (30 mg/kg every two days for three weeks), and the heart, liver, spleen, lung, and kidney were harvested for H&E staining. ICR/JCL mice were intraperitoneally injected with M1-21 (100 mg/kg n = 3, 150 mg/kg n = 3, and 200 mg/kg n = 3) and continuously observed for diet and activity for 14 days to investigate acute toxicity of M1-21.

### Hemolysis and immunogenicity assays of M1-21

Hemolysis assays were performed with fresh mouse blood collected and dispersed in normal saline (NaCl 0.9%) at 10% (v/v). The suspended erythrocytes (100 µL per sample) were treated with different concentrations of M1-21 (0, 25, 50, 100, 200, 400, 800 µg/mL) at 37℃ for 3 h. Sterile double distilled water and PBS were used as positive and negative controls, respectively. Immunogenicity of M1-21 was detected by ELISA in mice. 6 weeks old female ICR/JCL mice (n = 3) were selected and injected intraperitoneally with M1-21 (30 mg/kg) once a week for 4 weeks. Blood samples were collected from mice at different time points (Week 2 to Week 8). The M1-21 peptide was dissolved in the coating buffer and coated at a concentration of 1 µg/well for 12 h at 4 °C. ELISA plates were washed twice with PBS and blocked with 3% bovine serum albumin for 2 h. After washing twice with PBS, the collected serum of mice from week 1 to 8 was diluted 1:1000 to measure the change in serum anti-M1-21 antibody concentration. HRP-conjugated anti-mouse IgG was used as an ELISA secondary antibody. Absorption was measured at 492 nm.

### Statistical analysis

We calculated three replicates between samples using Microsoft Excel or GraphPad Prism 8.0. Briefly, we calculated replicates in the control and experimental groups. Using GraphPad Prism software, Kolmogorov-Smirnov (distance) and Shapiro-Wilk (W) were used to test the normal distribution of data. Subsequently, the unpaired t-test was performed for each sample value between the control and experimental groups. *P < 0.05; **P < 0.01; ***P < 0.001. *P* < 0.05 was considered to indicate a statistically significant difference.

## Electronic supplementary material

Below is the link to the electronic supplementary material.

Supplementary Material 1

## Data Availability

Data sets used and/or analyzed during the current study are available from the corresponding author upon reasonable request.
