# Supplementary material for: The development of an anti-cancer peptide M1-21 targeting transcription factor FOXM1
Source: Cell Biosci. 2023 Jun 21;13:114. doi: 10.1186/s13578-023-01059-7 (PMC10286459; doi:10.1186/s13578-023-01059-7)

**The development of an anti-cancer peptide M1-21 targeting transcription factor FOXM1**

Table of Contents

**Supplementary figures**4

Figure S1. Analysis of peptide TAT-96-116 by MALDI TOF MS and HPLC4

Figure S2. Analysis of peptide TAT-101-121 by MALDI TOF MS and HPLC5

Figure S3. Analysis of peptide TAT-106-126 by MALDI TOF MS and HPLC6

Figure S4. Analysis of peptide TAT by MALDI TOF MS and HPLC7

Figure S5. Interaction assays of biotin-labeled peptides TAT-96-116, TAT-101-121 and TAT-106-126 with FOXM1 (688-748) protein, and FOXM1 full-length protein8

Figure S6. Comparison of sequences and branched chain structures of TAT-106-126 and M1-219

Figure S7. Analysis of peptide M1-21 by MALDI TOF MS and HPLC10

Figure S8. TAT-106-126 and M1-21 bind to human FOXM1 protein11

Figure S9. Analysis of peptide M1-21mut by MALDI TOF MS and HPLC12

Figure S10. M1-21mut loses ability to bind FOXM1_688-748_ or inhibit cancer cells 13

Figure S11. M1-21 did not inhibit non-tumor MCF-10A cells14

Figure S12. M1-21 decreased the levels of FOXM1, CDC25B, and PLK1 in nude MDA-MB-231 cell-engrafted mice15

Figure S13. M1-21mut did not bind to FOXM1_1-138_ and FOXM1_232-332_ proteins16

Figure S14. M1-21 did not disrupt FOXM1's binding to DNA17

Figure S15. Construction of 231-Flag-FOXM1-Ind cell line18

Figure S16. TAT-106-126 disrupted FOXM1's interaction with PLK119

Figure S17. M1-21 inhibited the WNT signaling pathway in MDA-MB-231 cells20

Figure S18. M1-21 bound to mouse Foxm1 and inhibited mouse breast cancer 4T1 cells21

Figure S19. Monitoring M1-21-treated mice with spontaneous breast cancers22

Figure S20. Construction of 4T1-Luc-GFP cells23

Figure S21. M1-21 downregulated FOXM1 mRNA and protein levels in cancer cells and tissues24

**Supplementary methods**25

Construction of plasmids25

Expression and purification of recombinant proteins28

Protein extraction and Western blotting29

Quantitative real-time PCR (qPCR)30

Cell viability, Colony formation, and Wound healing assays30

Pulldown and Co-immunoprecipitation assays31

Luciferase activity assays31

Electrophoretic mobility shift assays (EMSAs)32

Immunohistochemistry32

**References**33

**Supplementary tables**34

Supplementary table S134

Supplementary table S236

Supplementary table S337

**Original WB data**38

**Supplementary Figures**

**
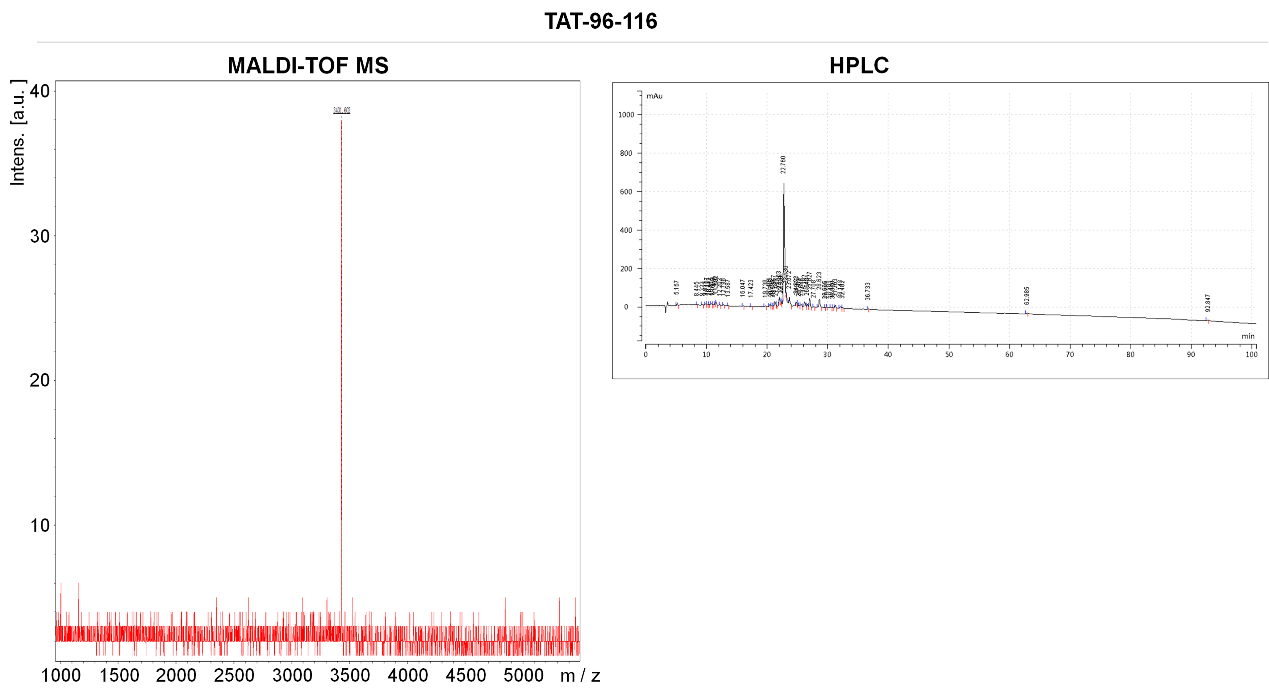
**

**Figure S1. Analysis of peptide TAT-96-116 by MALDI TOF MS and HPLC.**

The molecular weight of the peptide was identified using Matrix-Assisted Laser Desorption/Ionization Time of Flight Mass Spectrometry (UltrafleXtreme, BRUKER, Germany). The purity of the peptide was determined by High Performance Liquid Chromatography (LC-2010&LC1010, RAINBOW, China) with acetonitrile and water (0.05%TFA) as mobile phase and C18 column (kromasil 100-5 C18 250*4.6*5, Sweden). At elution time (100 min), the elution gradient (0.5% to 70% acetonitrile) was set for separation of the peptide.

**
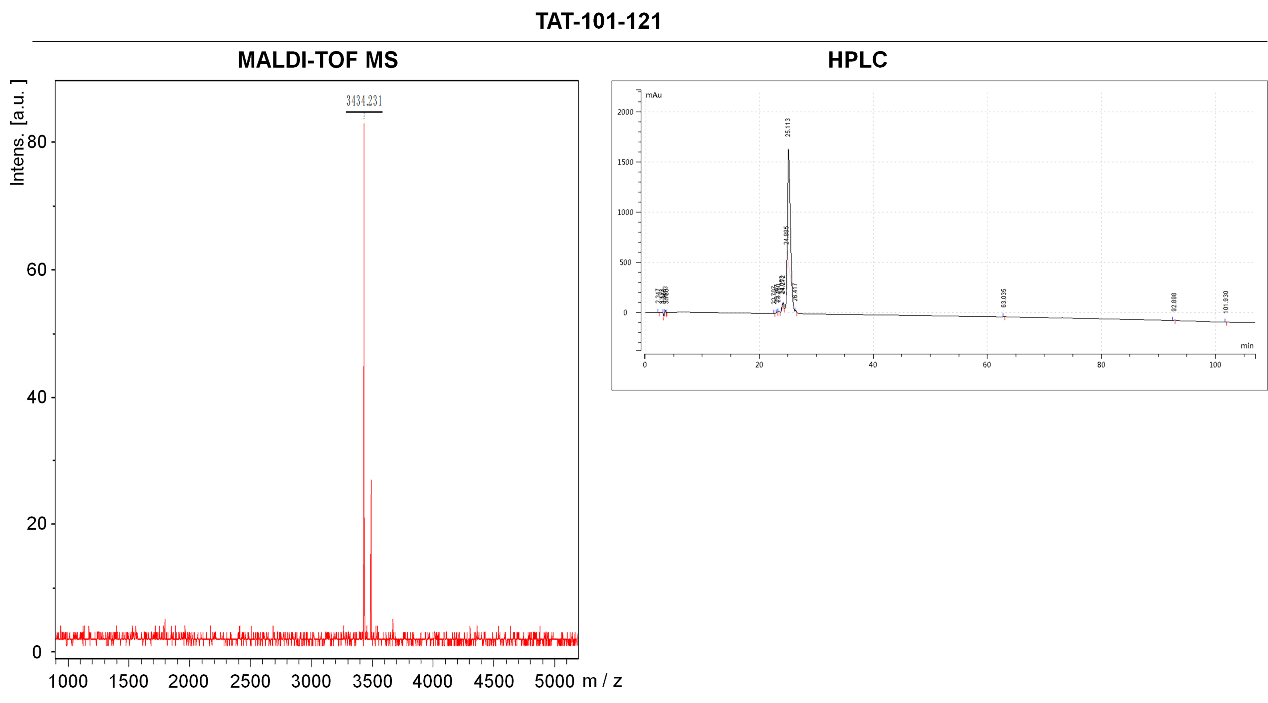
**

**Figure S2. Analysis of peptide TAT-101-121 by MALDI TOF MS and HPLC.**

The methods were the same as in Figure S1.

**
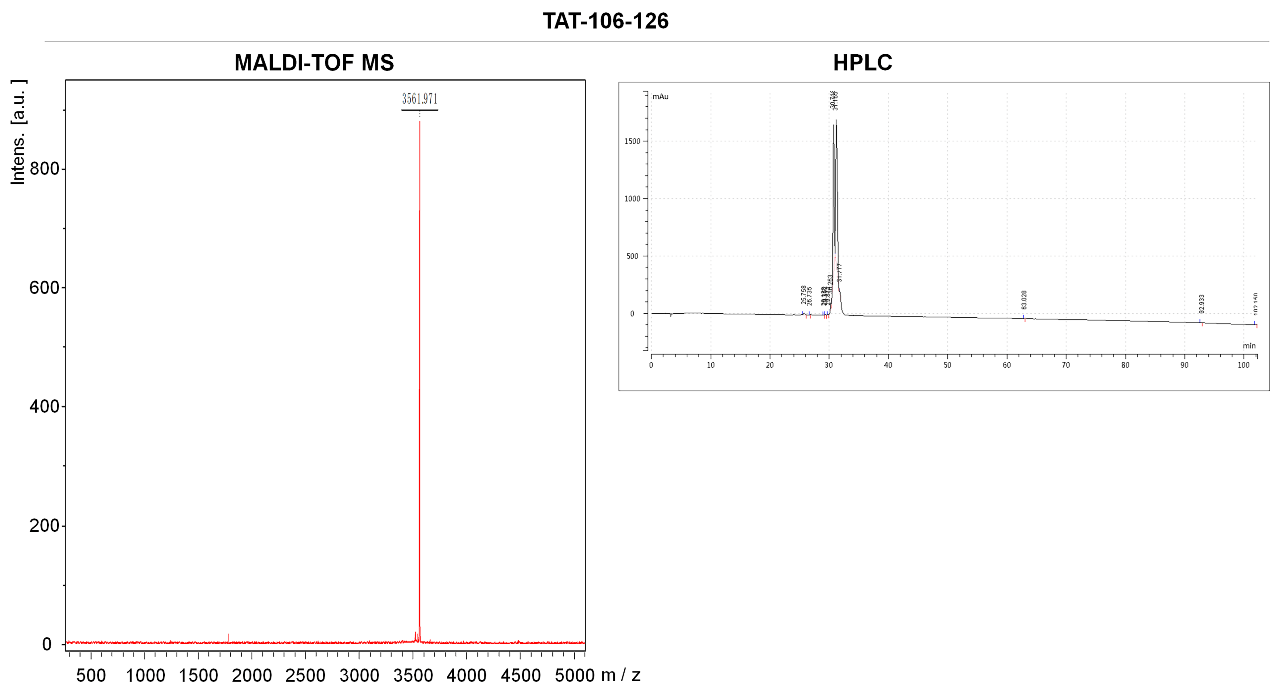
**

**Figure S3. Analysis of peptide TAT-106-126 by MALDI TOF MS and HPLC.**

The methods were the same as in Figure S1.

**
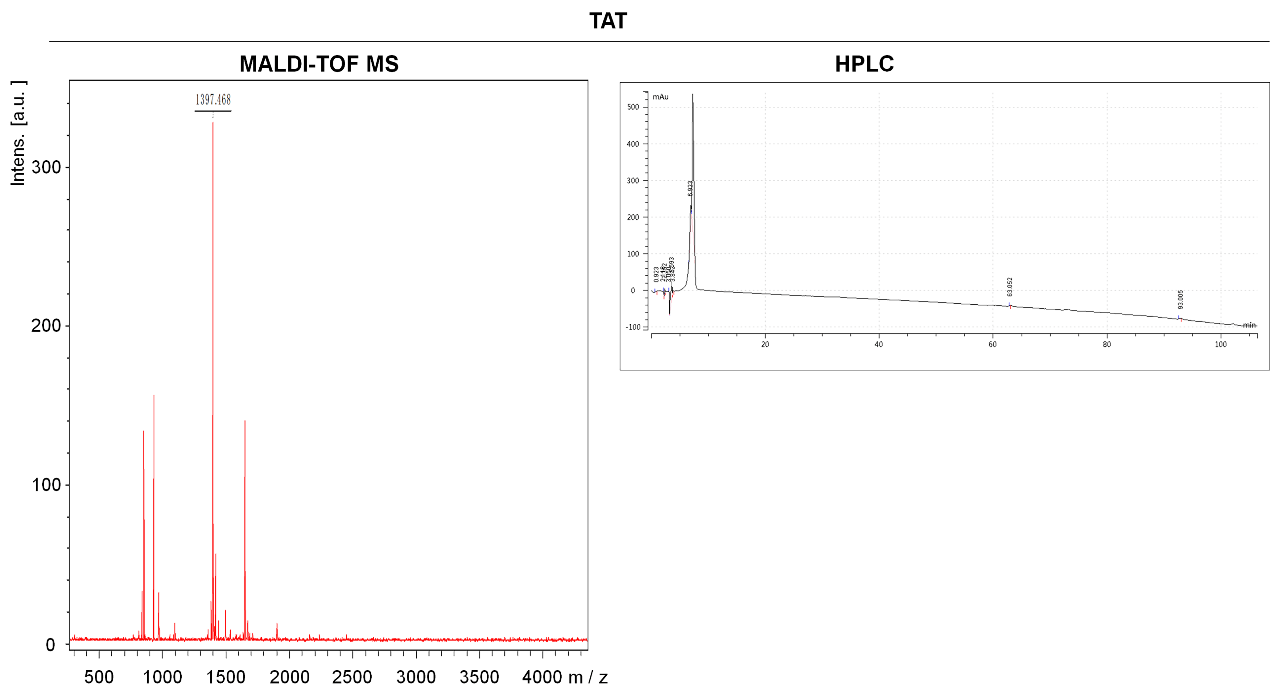
**

**Figure S4. Analysis of peptide TAT by MALDI TOF MS and HPLC.**

The methods were the same as in Figure S1.





**Figure S5. Interaction assays of biotin-labeled peptides TAT-96-116, TAT-101-121, and TAT-106-126 with FOXM1 (688-748) protein, and FOXM1 full-length protein.**

**A** Biotin-labeled peptides TAT-96-116, TAT-101-121, and TAT-106-126 were added to 293T cell lysates (500 μg) expressing exogenous Flag-GFP-FOXM1(688-748) protein (or Flag-GFP control lysates). The lysates were incubated with Streptavidin magnetic beads to pull down biotin-peptide/protein complexes. Biotin and Flag-GFP-FOXM1(688-748) protein in samples were detected by Western Blotting. 5% of cell lysates (25 μg) were used as input controls. **B** Biotin-labeled TAT-96-116, TAT-101-121, TAT-106-126, and TAT were added to 293T cell lysates (500 μg) expressing exogenous FOXM1 protein. The lysates were incubated with Streptavidin magnetic beads to pull down biotin-peptide/protein complexes. Biotin and exogenous FOXM1 protein were detected by Western Blotting. 5% of cell lysates (25 μg) were used as input controls.

**

**

**Figure S6. Comparison of sequences and branched chain structures of TAT-106-126 and M1-21**.

**
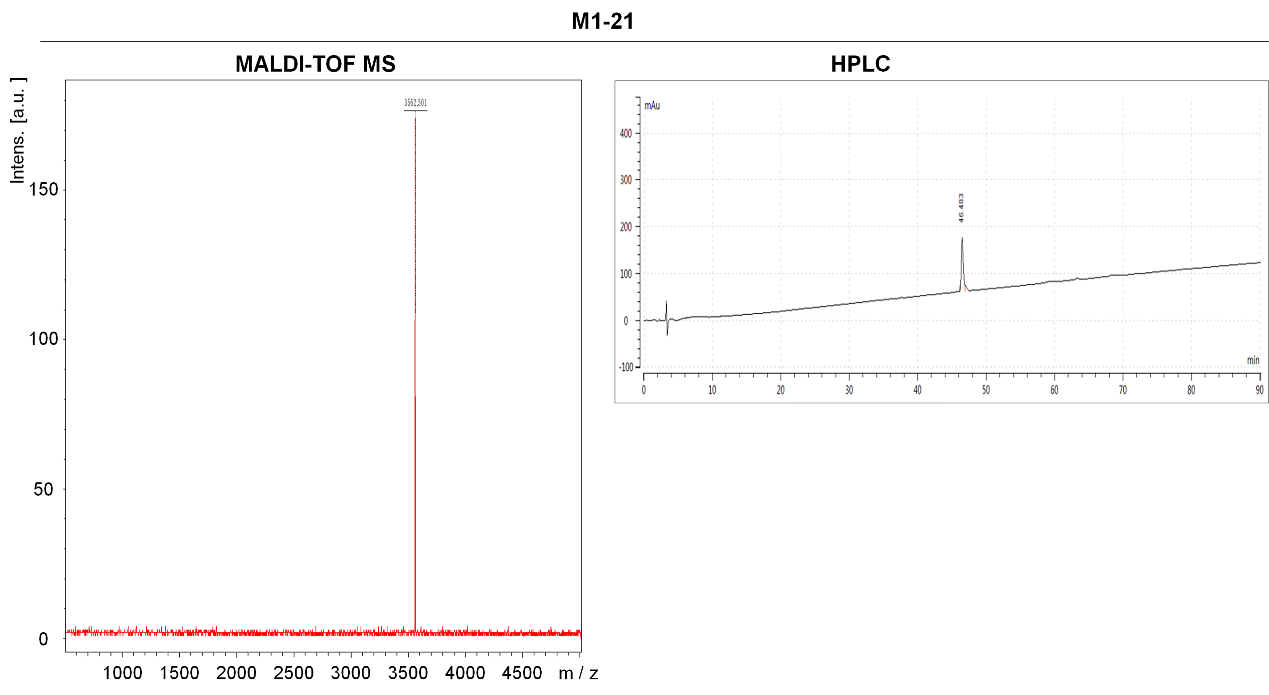
**

**Figure S7. Analysis of peptide M1-21 by MALDI TOF MS and HPLC.**

The methods were the same as in Figure S1.


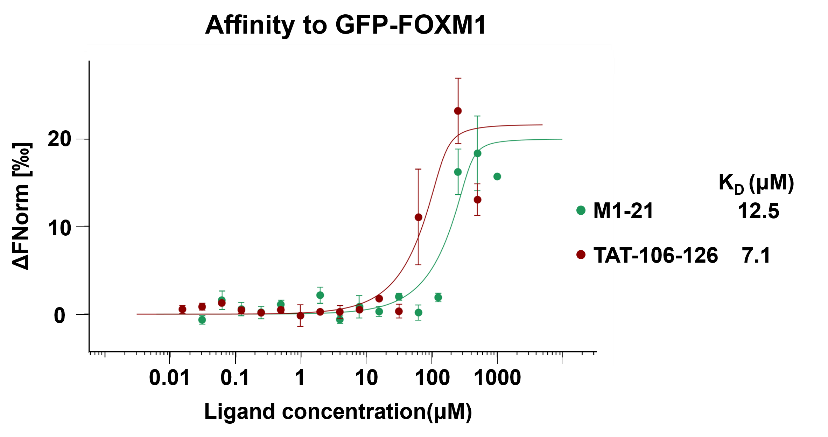


**Figure S8. TAT-106-126 and M1-21 bind to human FOXM1 protein.**

The binding affinity between human FOXM1 protein (GFP-FOXM1) and TAT-106-126, M1-21 was quantified by MST. Experiments were repeated three times with similar results.

**
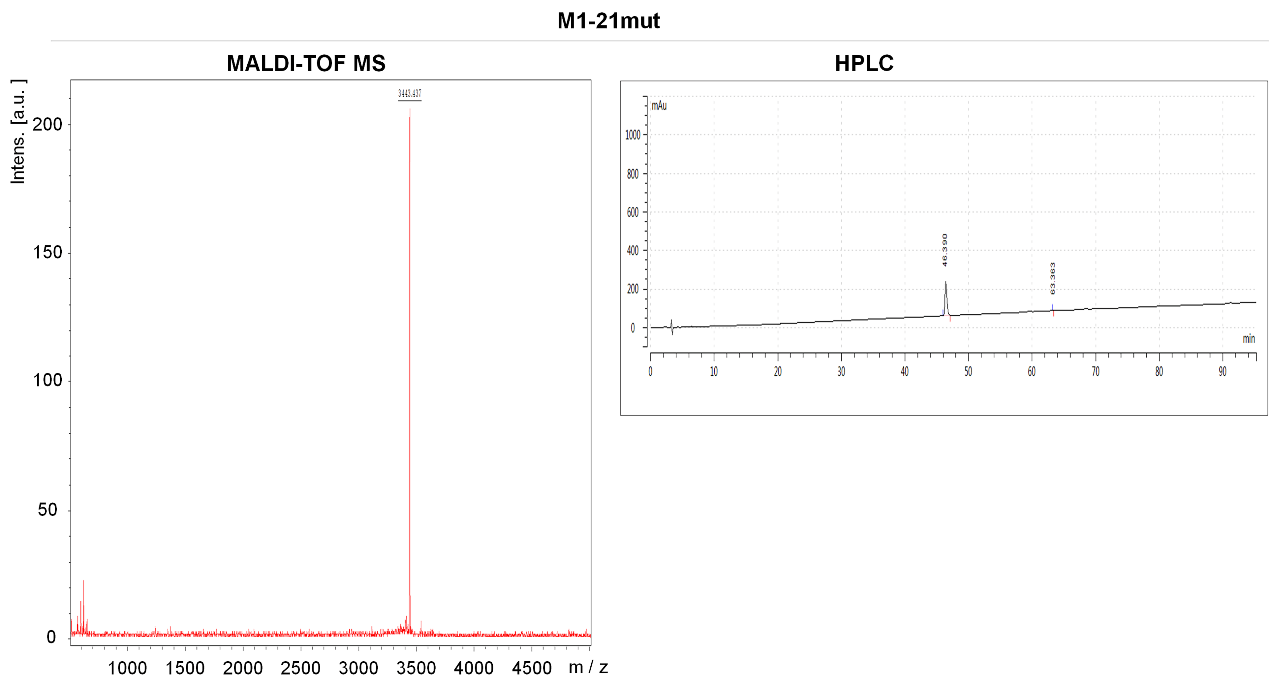
**

**Figure S9. Analysis of peptide M1-21mut by MALDI TOF MS and HPLC.**

The methods were the same as in Figure S1.

**
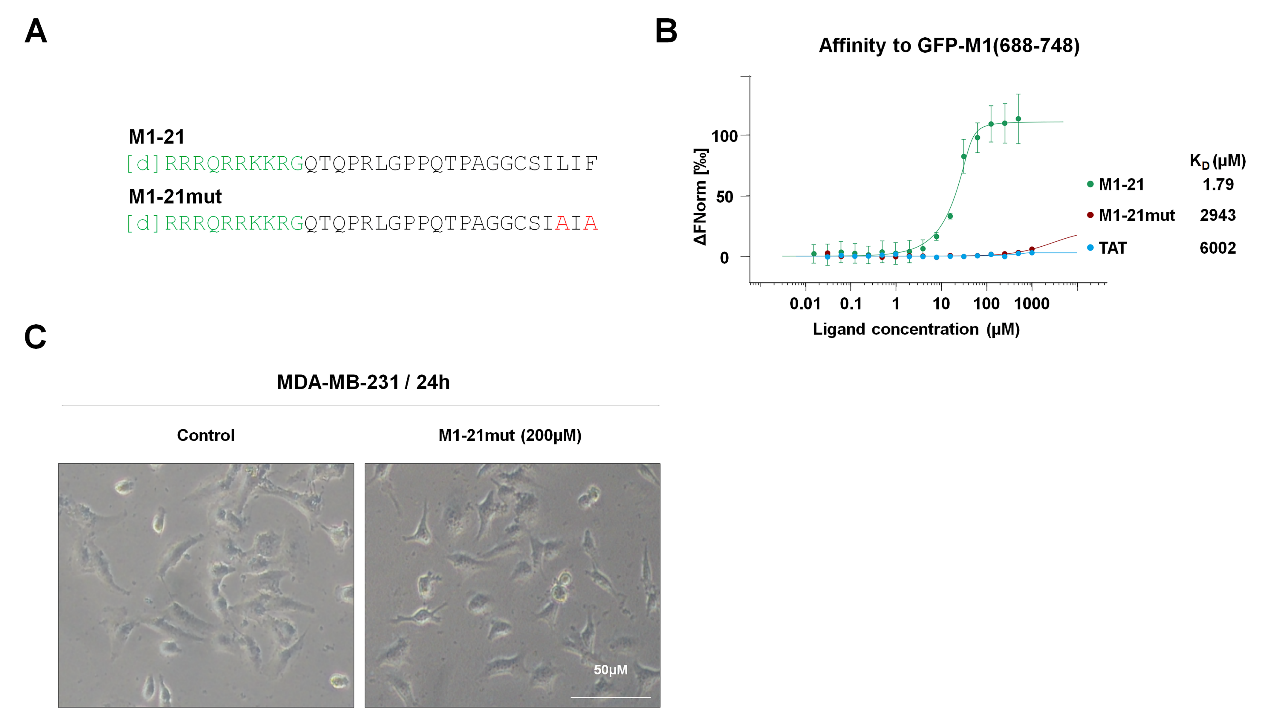
**

**Figure S10. M1-21mut loses ability to bind FOXM1_688-748_ or inhibit cancer cells.**

**A** Comparison between M1-21 and M1-21mut sequences. **B** Quantification of binding affinity between GFP-FOXM1_688-748_ (GFP-M1(688-748)) and M1-21, M1-21mut, or TAT was performed by MST. Experiments were repeated three times with similar results. **C** M1-21mut did not inhibit cancer cells. MDA-MB-231 cells were treated with M1-21mut (200 μM) for 24 hours and the image was recorded by microscope imaging (200×, Nikon TE2000). Scale bar: 50 μm.

**

**

**Figure S11. M1-21 did not inhibit non-tumor MCF-10A cells.**

MCF-10A cells (2×10^5 cells/well) were seeded in 24-well plates for 12 h and treated with different concentrations of M1-21 or M1-21mut (0, 10, 20, 30, 40, 50 µM). 72 h Later, trypan blue staining (0.4%) was added to each well and incubated for another 3 min and the cells were fixed at 4% paraformaldehyde for imaging. The number of viable cells was counted by ImageJ software to calculate the cell viability of each well. The correspondence between cell viability and peptide concentration was plotted by GraphPad software.


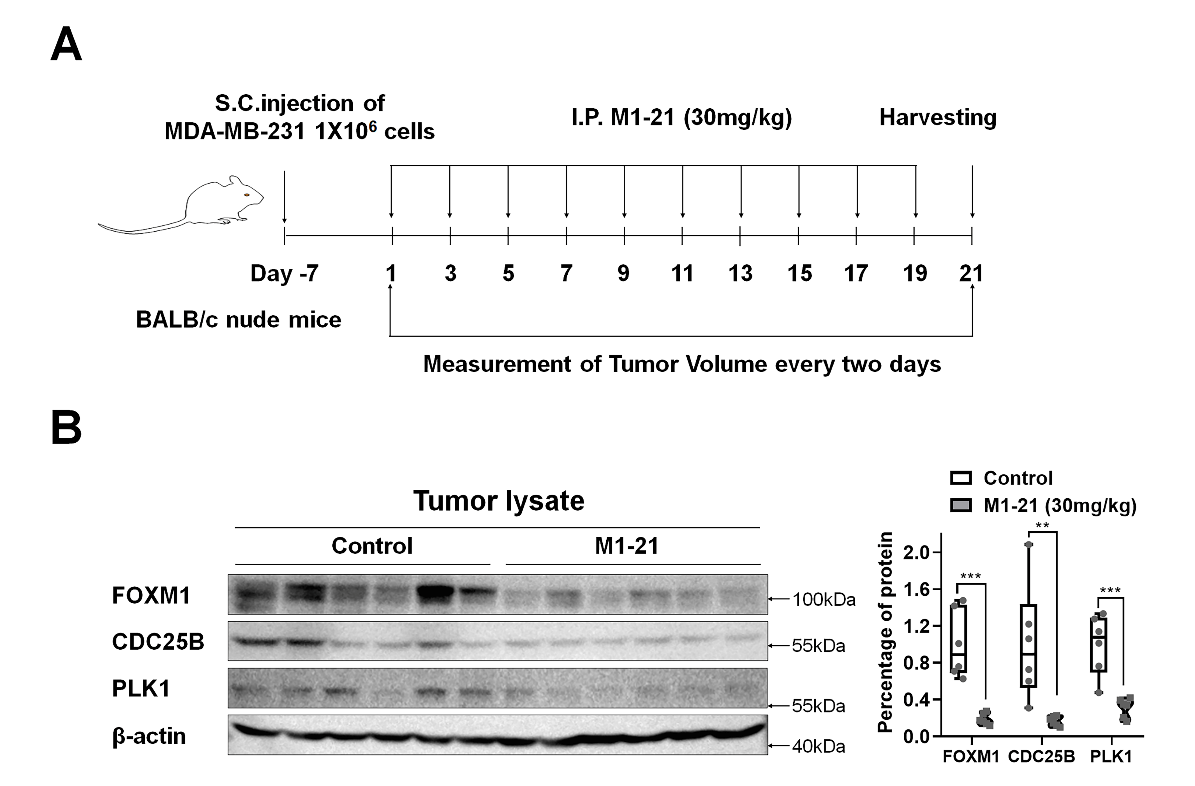


**Figure S12. M1-21 decreased the levels of FOXM1, CDC25B, and PLK1 in nude MDA-MB-231 cell-engrafted mice.**

**A** Method for generating BALB/c nude mice with MDA-MB-231 cells and treating M1-21. **B** The lysates of the harvested cancer tissues were obtained and the protein levels of FOXM1, CDC25B, and PLK1 were detected by Western blotting. β-actin was used as a loading control. ImageJ software was used to calculate the gray value of the bands, and each point represented the value of protein content in the samples.

**
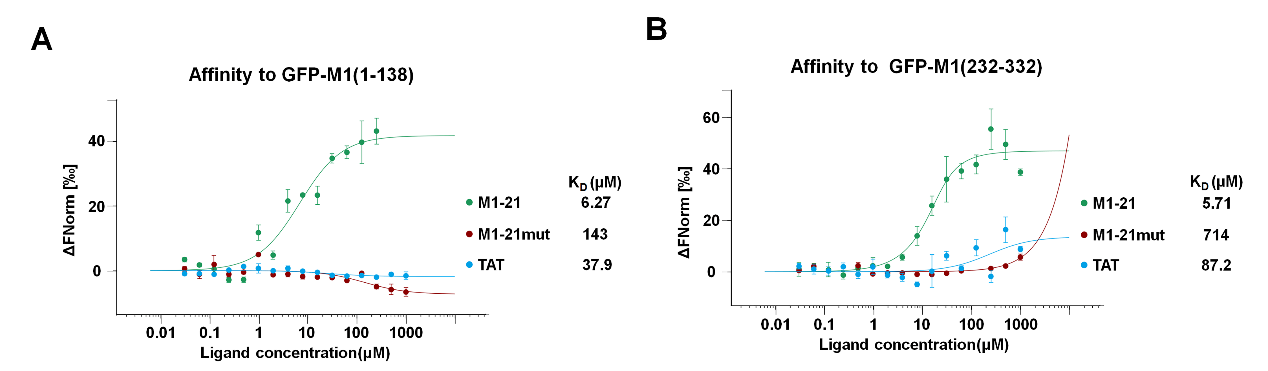
**

**Figure S13.** **M1-21mut did not bind to FOXM1_1-138_ and FOXM1_232-332_ proteins.**

Quantification of binding affinity between GFP-FOXM1_1-138_ (GFP-M1(1-138)) and M1-21, M1-21mut, or TAT (**A**), or between GFP-FOXM1_232-332_ (GFP-M1(232-332)) and M1-21, M1-21mut, or TAT (**B**) was performed by MST. Experiments were repeated three times with similar results.

**
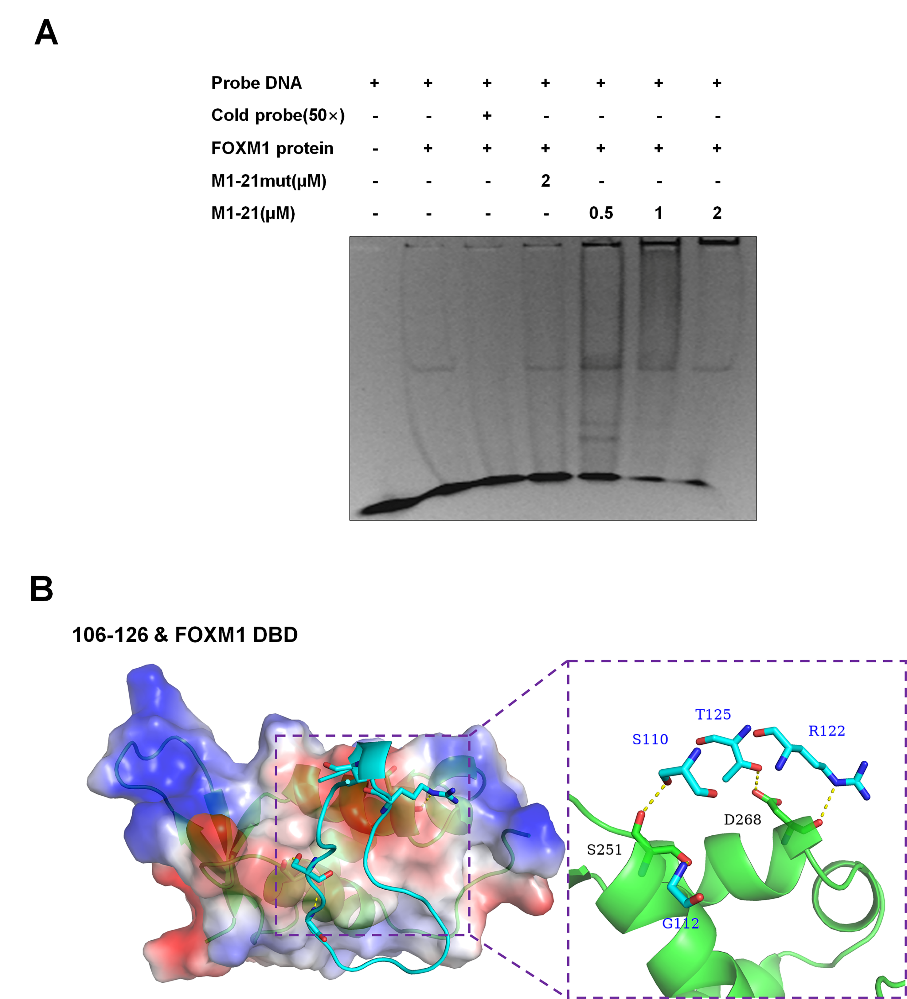
**

**Figure S14. M1-21 did not disrupt FOXM1's binding to DNA.**

**A** FAM-labeled DNA probe (50 nM) was mixed with recombinant FOXM1 protein (2 μM) for EMSAs. M1-21mut (2 μM) or .M1-21 was added to the reaction at increasing concentrations (0.5, 1, and 2 μM). The specificity of FOXM1/DNA complex formation was shown using 100× unlabeled DNA probes (cold probes, 5000 nM). Reactions were resolved in 4% native polyacrylamide gel electrophoresis in 0.5×TBE and visualized with a Kodak 4000 MM Imaging System (EX: 465 nm, EM: 535 nm). **B** The docking of P22 to FOXM1 DNA Binding domain (DBD, PDB ID 3G73) was created by Rosetta FlexPepDock and PyMOL. Left, the interface of peptide-protein interaction, peptide was shown in cyan and FOXM1 DBD was shown according to its electrostatic potential. Right, hydrogen bonds were formed by residues (blue from peptide and black from FOXM1 DBD) at the interface of peptide and protein interaction.





**Figure S15. Construction of 231-Flag-FOXM1-Ind cell line.**

**A** MDA-MB-231 cells were infected by lentivirus containing TetOne-Flag-FOXM1B expression cassette and the stable cell line was selected by puromycin and named 231-Flag-FOXM1-Ind. The cells were treated with a Doxycycline concentration gradient (0, 5, 10, 50, 200, 500 ng/mL) for 12 h and lysates were extracted to detect Flag-FOXM1, FOXM1, and β-actin levels by Western blotting. **B** Panel A lysates (200 ng/ml) were used to detect FOXM1, CDC25B, and PLK1 levels by Western blotting. β-actin was used as a loading control.





**Figure S16. TAT-106-126 disrupted FOXM1's interaction with PLK1.**

The Flag-FOXM1 inducible MDA-MB-231 cell line (231-Flag-FOXM1-Ind) was induced with doxycycline (200 ng/mL) for 24 h and then treated with M1-21mut (20 μM) or TAT-106-126 (20, 40, 80 μM) for 6 h. The cell lysates (500 μg) were extracted and incubated with the Anti-Flag Magnetic Beads to pull down the Flag-FOXM1/proteins complexes. The levels of Flag-FOXM1 and PLK1 proteins in the samples were detected by Western blotting. 5% of cell lysates (25 μg) were used as input controls.

**
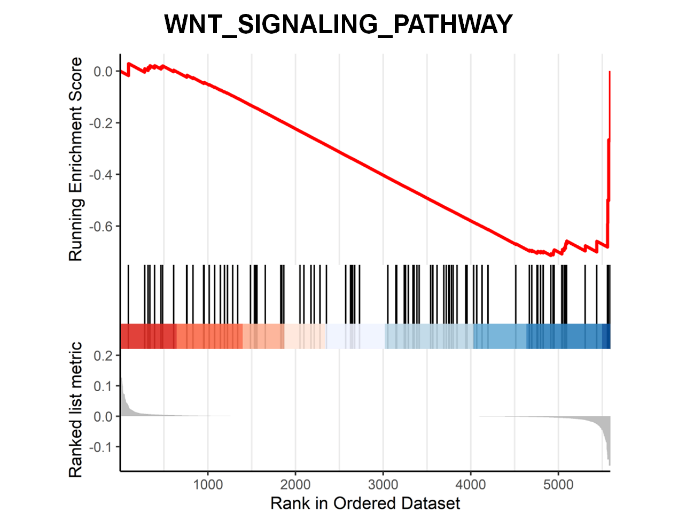
**

**Figure S17. M1-21 inhibited the WNT signaling pathway in MDA-MB-231 cells.**

MDA-MB-231 cells were treated with M1-21 or M1-21mut (20 μM) for 24 h and total RNA samples were prepared for RNA sequencing. Gene set enrichment analysis (GSEA) was performed to show inhibition of genes in the WNT signaling pathway.


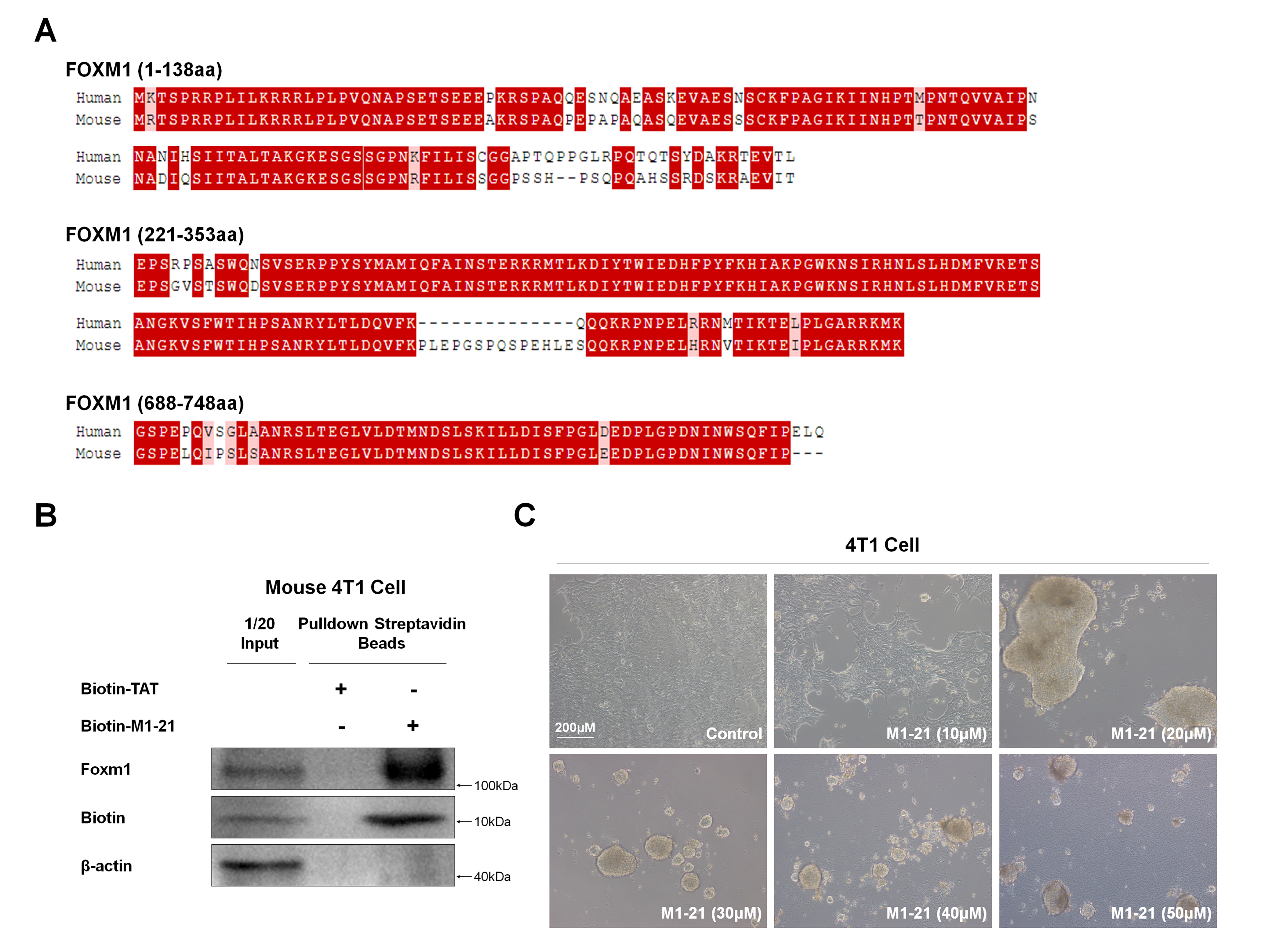


**Figure S18. M1-21 bound to mouse Foxm1 and inhibited mouse breast cancer 4T1 cells.**

**A** The alignment between different regions (1-138aa), (221-353aa), and (688-748aa) of human FOXM1 and the corresponding Foxm1 mouse regions. **B** Biotin-labeled TAT (Biotin-TAT, 20 μM) or M1-21 (Biotin-M1-21, 20 μM) was added to 4T1 cell lysates (500 μg). Streptavidin magnetic beads were added to lysates to pull down Biotin-peptide/protein complexes detected by Western blotting. 5% of cell lysates (25 μg) were used as input controls. **C** 4T1 cells were treated with gradient concentrations of M1-21 (0, 10, 20, 30, 40, 50 μM) for 24 h and then stained with trypan blue (0.4%) for 3 min. Cell imaging was performed by microscope imaging (200×, Nikon TE2000). Scale bar: 200 μm.

**
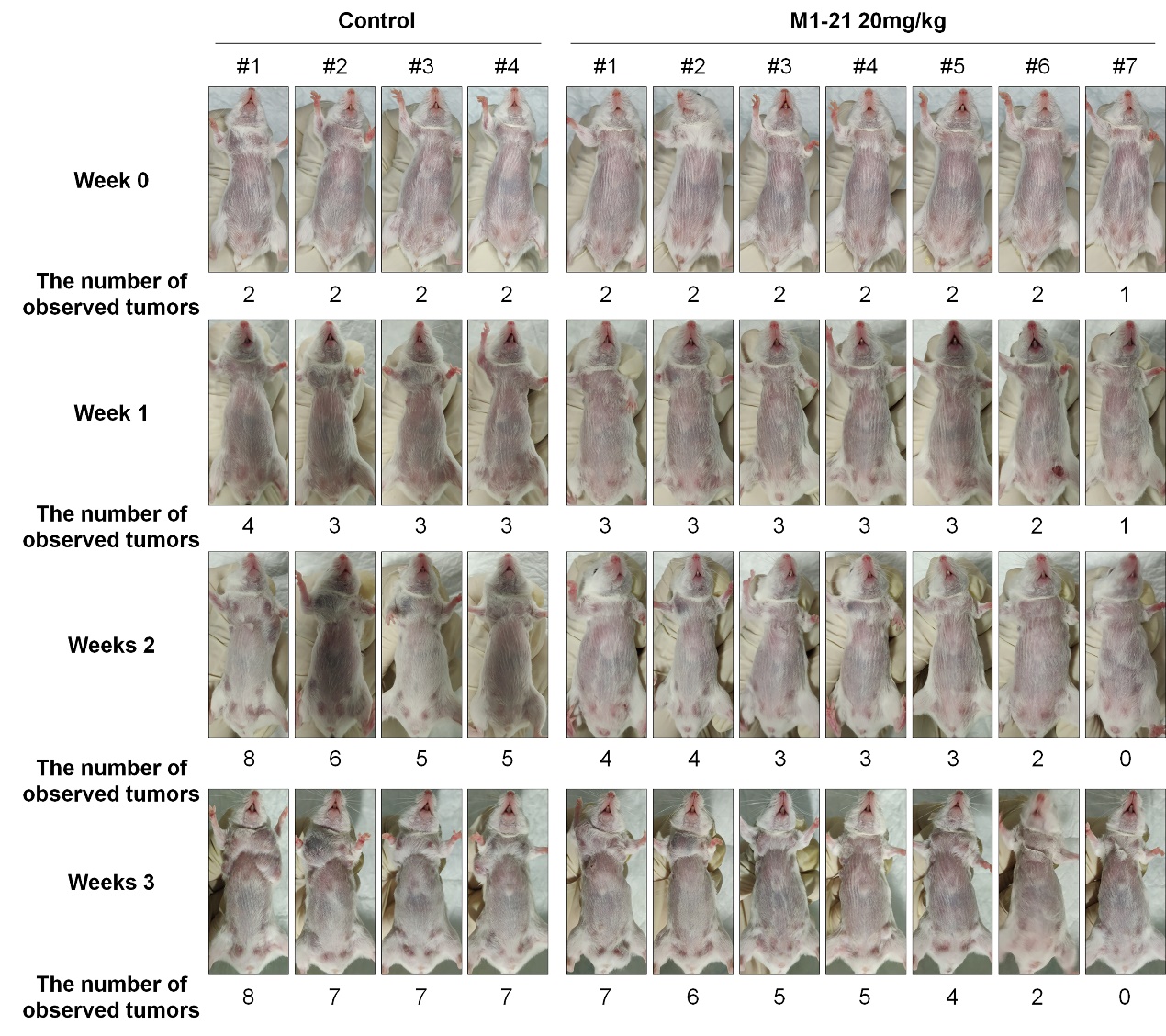
**

**Figure S19. Monitoring M1-21-treated mice with spontaneous breast cancers.**

FVB/N MMTV-PyVT mice (female, 8 weeks old, beginning to form spontaneous breast cancer) were injected intraperitoneally with PBS (Control, n = 4) or M1-21 (20 mg/kg, n = 7) once daily for 28 days. The mice were imaged at different time points after treatment (Week 0, 1, 2, and 3). The number of tumors observed in each mouse was counted.

**
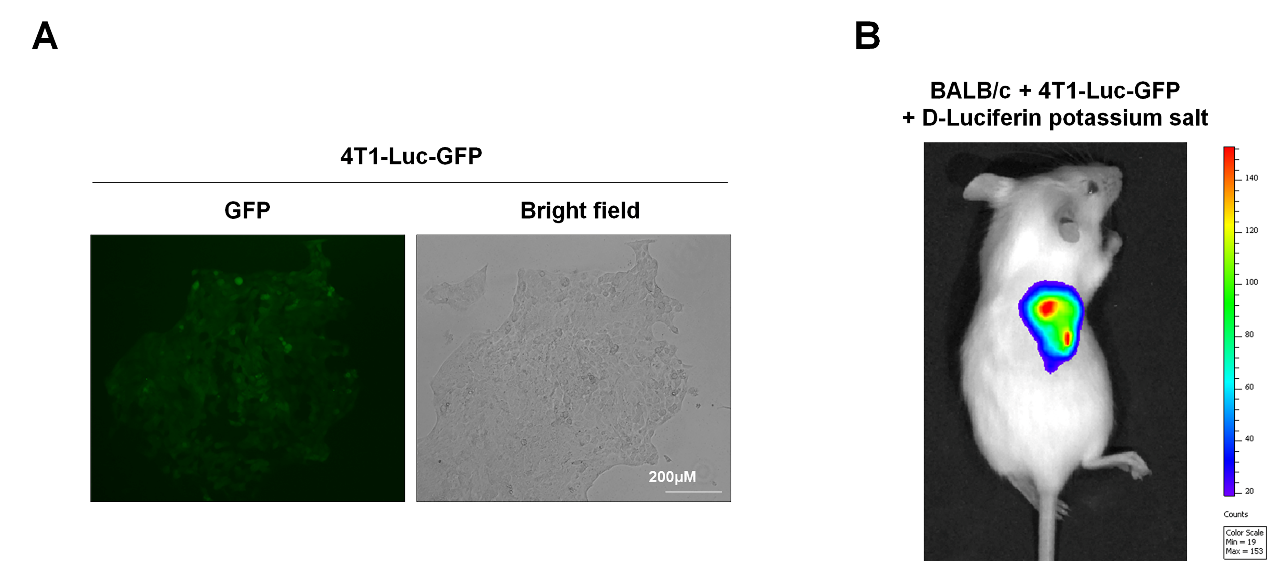
**

**Figure S20. Construction of 4T1-Luc-GFP cells.**

**A** 4T1 cells were infected with lentivirus containing the luciferase-GFP expression cassette and the stable cell line was selected by puromycin and named 4T1-Luc-GFP. The expression of GFP in the cells was detected by a florescent microscope (200×, OLYMPUS IX73). **B** 4T1-Luc-GFP cells (1×10^6 cells) were injected subcutaneously into wild-type BALB/c mice, and 7 days later, subjected to intraperitoneal injection of D-Luciferin potassium salt (3 mg/200 mL/mouse) which was dissolved in D-PBS and imaged using an IVIS Lumina XR machine.


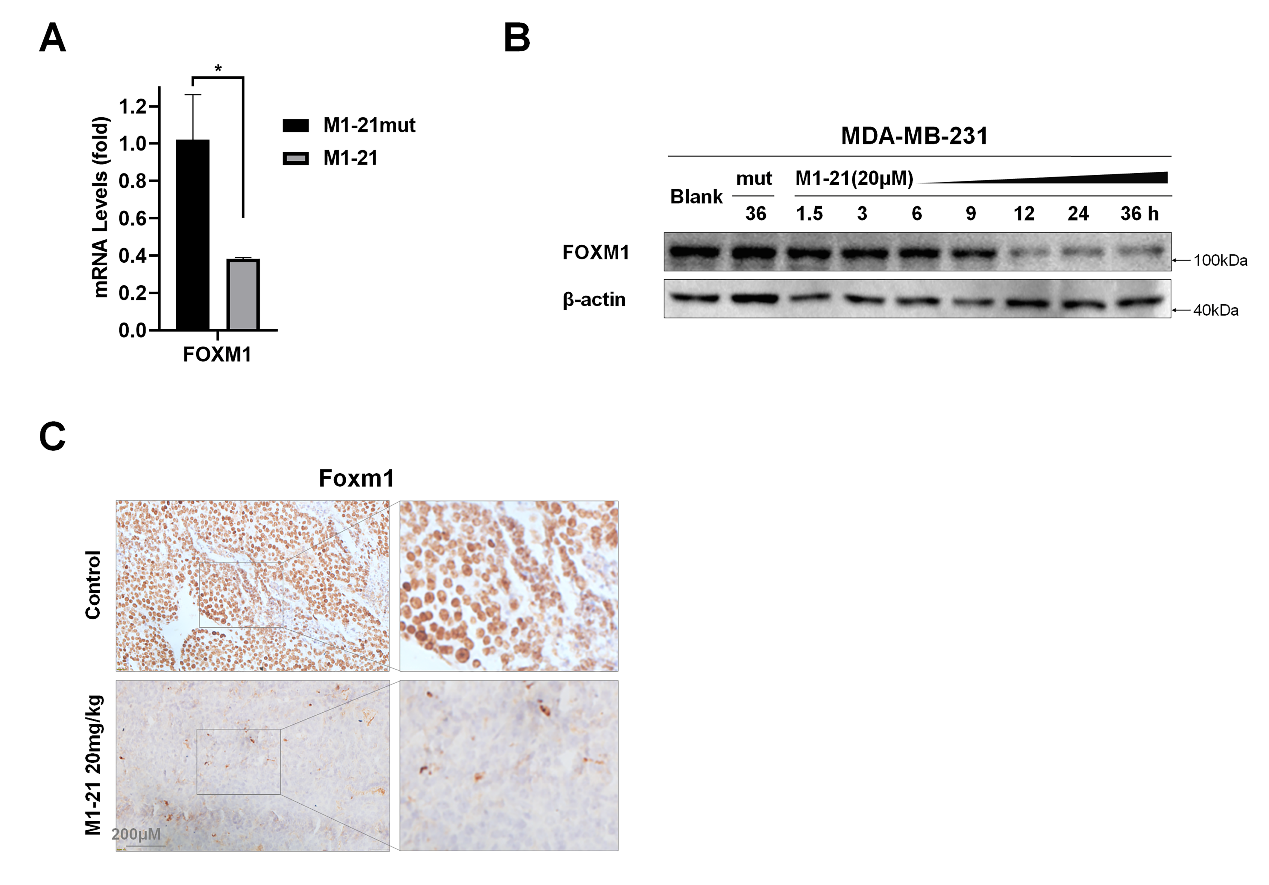


**Figure S21. M1-21 downregulated FOXM1 mRNA and protein levels in cancer cells and cancer tissues.**

**A** MDA-MB-231 cells were treated with M1-21mut or M1-21 (20 μM) for 24 h. FOXM1 mRNA was detected by qPCR. GAPDH was used as a loading control. **B** MDA-MB-231 cells were treated with M1-21mut (20 μM, 36 h) or M1-21 (20 μM) at different time points (1.5, 3, 6, 9, 12, 24, 36 h). The protein level of FOXM1 was measured by Western blotting. β-actin was used as a loading control. **C** The representative sections of the cancer samples in mice with spontaneous breast cancers were immunostained with anti-Foxm1 antibody (Santa Cruz, Cat#sc-376471, 1:200) followed by microscope imaging (200×, Nikon TE2000). The scale bar: 200 μm. mRNA values represented the mean ± SD of three replicates, and significance was calculated using unpaired *t* test. **P* < 0.05, ***P* < 0.01, ****P* < 0.001.

**Supplementary Methods**

**Construction of plasmids**

The cDNA of EGFP was PCR amplified from pEGFP-C2 plasmid (Addgene) with primers containing XhoI and BamHI restriction sites, and Flag-tag (sense primers: 5’-CCG CTC GAG ATG GAT TAC AAG GAT GAC GAC GAT AAG ATG GTG AGC AAG GGC GAG GA-3’ and antisense primers: 5’-CGG GAT CCT TAC TTG TAC AGC TCG T-3’) and ligated into the pcDNA-3.1 vector (Thermo Fisher Scientific, USA) to obtain the plasmid pcDNA3.1-Flag-GFP. The cDNA of FOXM1(688-748aa) was PCR amplified from pCMV-FOXM1 plasmid[1] with primers containing XhoI and BamHI restriction sites, and Flag-tag (sense primers: 5’-CCG CTC GAG ATG GAT TAC AAG GAT GAC GAC GAT AAG GGC TCC CCG GAG CCA CAG GT-3’ and antisense primers: 5’-CGG GAT CCC TAC TGT AGC TCA GGA ATA A-3’) and ligated into the pcDNA-3.1 vector to obtain the plasmid pcDNA3.1-Flag-M1(688-748). The cDNA of GST was PCR amplified from pET-Dual-N-GST plasmid (Beyotime, Cat# D2931-1μg) with primers containing NcoI and BamHI restriction sites (sense primers: 5’-GCG CCC ATG GAT GGG CTC CCC TAT ACT AGG-3’ and antisense primers: 5’-CGG GAT CCT TAT TTT GGA GGA TGG TCG CCA C-3’) and ligated into the pET-15b vector (Novagen, Germany) to obtain the plasmid pET-15b-GST. A pET-15b-GST (TAA-deficient) plasmid lacking a stop codon was cloned synchronously by using antisense primers 5’-CGG GAT CCT TTT GGA GGA TGG TCG CCA C-3’. The cDNA of FOXM1(1-138aa) was PCR amplified from a pCMV-FOXM1 plasmid with primers containing BamHI restriction sites (sense primers: 5’-GTG GCG ACC ATC CTC CAG GAT CCA AAA TGA AAA CTA GCC CCC GTC G-3’ and antisense primers: 5’-CGG GCT TTG TTA GCA GCC GGA TCC TTA CAG GGT CAC TTC TGT CCT TT-3’) and homologous recombination (TaKaRa, Japan) ligated into the pET-15b-GST (TAA-deficient) vector to obtain the plasmid pET-15b-GST-M1(1-138). The cDNA of FOXM1(221-353aa) was PCR amplified from a pCMV-FOXM1 plasmid with primers containing BamHI restriction sites (sense primers: 5’-GTG GCG ACC ATC CTC CAA AAG GAT CCG AGC CTT CGA GAC CAT CAG C-3’ and antisense primers: 5’-CGG GCT TTG TTA GCA GCC GGA TCC TTA CTT CAT CTT CCG CCG TGC-3’) and homologous recombination ligated into the pET-15b-GST (TAA-deficient) vector to obtain the plasmid pET-15b-GST-M1(221-353). The cDNA of FOXM1(330-520aa) was PCR amplified from a pCMV-FOXM1 plasmid with primers containing BamHI restriction sites (sense primers: 5’-GTG GCG ACC ATC CTC CAA AAG GAT CCC CGA ATC CAG AGC TCC GCC G-3’ and antisense primers: 5’-CGG GCT TTG TTA GCA GCC GGA TCC TTA GTG TTG AAT CAC AAG CAT TT-3’) and homologous recombination ligated into the pET-15b-GST (TAA-deficient) vector to obtain the plasmid pET-15b-GST-M1(330-520). The cDNA of FOXM1(500-680aa) was PCR amplified from a pCMV-FOXM1 plasmid with primers containing BamHI restriction sites (sense primers: 5’-GTG GCG ACC ATC CTC CAA AAG GAT CCA AGT CCT ACA GTG GGC TTA G-3’ and antisense primers: 5’-CGG GCT TTG TTA GCA GCC GGA TCC TTA TGA GGG AGA AGA GTT GCC AA-3’) and homologous recombination ligated into the pET-15b-GST (TAA-deficient) vector to obtain the plasmid pET-15b-GST-M1(500-680). The cDNA of FOXM1(688-748aa) was PCR amplified from a pCMV-FOXM1 plasmid with primers containing BamHI restriction sites (sense primers: 5’-GTG GCG ACC ATC CTC CAA AAG GAT CCG GCT CCC CGG AGC CAC AGG T-3’ and antisense primers: 5’-CGG GCT TTG TTA GCA GCC GGA TCC CTA CTG TAG CTC AGG AAT AAA CT-3’) and homologous recombination ligated into the pET-15b-GST (TAA-deficient) vector to obtain the plasmid pET-15b-GST-M1(688-748). The cDNA of EGFP was PCR amplified from pEGFP-C2 plasmid (Addgene, USA) with primers containing EcoRI restriction sites (sense primers: 5’-GGC TGA TAT CGG ATC CGA ATT CAT GGT GAG CAA GGG CGA G -3’ and antisense primers: 5’-CTT GTC GAC GGA GCT CGA ATT CTT ACT TGT ACA GCT CGT C-3’) and homologous recombination ligated into the pET-32a (Novagen, Germany) vector to obtain the plasmid pET-32a-GFP. The cDNA of FOXM1(1-138aa) was PCR amplified from a pCMV-FOXM1 plasmid with primers containing BamHI restriction sites (sense primers: 5’-GGC CAT GGC TGA TAT CGG ATC CAT GAA AAC TAG CCC CC-3’ and antisense primers: 5’-GAC GGA GCT CGA ATT CGG ATC CCA GGG TCA CTT CTG TC-3’) and homologous recombination ligated into the pET-32a-GFP (We cloned it earlier) vector to obtain the plasmid pET-32a-GFP-M1(1-138). The cDNA of FOXM1(232-332aa) was PCR amplified from a pCMV-FOXM1 plasmid with primers containing BamHI restriction sites (sense primers: 5’-GGC CAT GGC TGA TAT CGG ATC CGA GCC TTC GAG ACC AT-3’ and antisense primers: 5’-GAC GGA GCT CGA ATT CGG ATC CCT TCA TCT TCC GCC GT -3’) and homologous recombination ligated into the pET-32a-GFP (We cloned it earlier) vector to obtain the plasmid pET-32a-GFP-M1(232-332). The cDNA of FOXM1(688-748aa) was PCR amplified from a pCMV-FOXM1 plasmid with primers containing BamHI restriction sites (sense primers: 5’-GGC CAT GGC TGA TAT CGG ATC CGG CTC CCC GGA GCC AC-3’ and antisense primers: 5’-GAC GGA GCT CGA ATT CGG ATC CCT GTA GCT CAG GAA TA-3’) and homologous recombination ligated into the pET-32a-GFP (We cloned it earlier) vector to obtain the plasmid pET-32a-GFP-M1(688-748). The cDNA of FOXM1 was PCR amplified from a pCMV-FOXM1 plasmid with primers containing AgeI restriction sites (sense primers: 5’-CGT AAA GAA TTC CCG TAT ACA CCG GAT GGA TTA CAA GGA TGA CGA CGA TAA GAA AAC TAG CCC CCG TCG GCC ACT GA-3’ and antisense primers: 5’-TCT GGA TCC GCC GGC ACC GGC TAC TGT AGC TCA GGA ATA A-3’) and homologous recombination ligated into the pLVX-TetOne-Puro vector (Biofeng, China) to obtain the plasmid pLVX-TetON-Flag-FOXM1b. The cDNA of Luc was PCR amplified from pGL3-Basic Vector plasmid (Promega, USA) with primers containing EcoRI and BamHI restriction sites (sense primers: 5’-GTG AGG ATC TAT TTC CGG TGA ATT CAT GGA AGA TGC CAA AAA CAT-3’ and antisense primers: 5’-GAG GGA GAG GGG CGG GAT CCT TAC ACG GCG ATC TTG CCG C-3’) and homologous recombination ligated into the pLVX-EF1α-IRES-EGFP vector (SiDanSai, China) to obtain the plasmid pLVX-EF1α-Luc-IRES-EGFP. pGL3-6×FOXM1 Binding-Luc, pGL3-*promoter*PLK1(-1.4kb)-Luc，and pGL3-*promoter*CDC25B(-1.8kb)-Luc reporter plasmids were described previously[2,3]. pGL3-TCF/LEF Binding-Luc was purchased from Yeasen Biotechnology Co., Ltd. (Shanghai, China).

**Expression and purification of recombinant proteins**

Certain plasmids were transformed into DE3 competent cells, and the transformed single colonies were identified by PCR. The single colony was inoculated into LB medium, and cultured at 37°C to absorbance 0.6-0.8 (OD600), followed by the addition of inducer 1 mM IPTG to induce protein expression for 24 hours at 28°C. GST, GST-M1(1-138), GST-M1(221-353), GST-M1(330-520), GST-M1(500-680), and GST-M1(688-748) recombinant proteins were purified according to the instructions of Glutathione Sepharose^TM^ 4B (GE, USA). GFP-M1(1-138), GFP-M1(232-332) and GFP-M1(688-748) recombinant proteins were purified according to the instructions of BeyoGold™ His-tag Purification Resin (Beyotime, China).

**Protein extraction and Western blotting**

The cells were washed with ice-cold 1×PBS and lysed on ice with IP lysate buffer containing 20 mM Tris/HCl pH 7.6, 150 mM NaCl, 1% NP-40, 0.1 mM EDTA, and protease inhibitors. Tumor tissues were homogenized using Tissue Lyser (Qiagen, Germany) and lysed in IP lysate buffer. After centrifugation, the protein concentration in the supernatant was measured with a BCA protein reagent (Thermo Fisher Scientific, USA). To extract cytoplasmic and nuclear proteins, cell pellets with 100 mm dish (> 90% confluence) were suspended in CE buffer (100 μL, 10 mM HEPES pH7.9, 1.5 mM MgCl2, 10 mM KCl, containing protease inhibitor) and incubated on ice for 5 min. An equal amount of CE buffer containing 0.2% NP40 was added to the cell suspension, incubated for 5 min on ice, and centrifuged for 3 min at 6500 rpm at 4℃. The supernatant is cytoplasmic extract. The pellet was resuspended in NE buffer (100 μL, 20 mM HEPES pH7.9, 1.5 mM MgCl2, 0.42 M NaCl, 0.2 mM EDTA, 25% glycerol, containing protease inhibitors) and vortexed at full speed for 1 min. The nuclear extract suspension underwent three cycles of freeze (-80℃, 15 min) and thaw (37 ℃, 1 min). Between each freeze/thaw cycle, the suspension was vortexed for 1 min at full speed. The suspension was then spun at full speed for 15 min at 4 ℃. The supernatant is nuclear extract. For Western blotting, samples were mixed with protein loading buffer containing beta-mercaptoethanol and heated to 95 ℃ for 10 min. Then, lysates were separated by SDS-PAGE gel electrophoresis and transferred to PVDF membranes, followed by Western blotting with certain antibodies. Antibody information is available in Supplementary Table S1.

**Quantitative real-time PCR (qPCR)**

Total RNA was extracted with an RNeasy mini kit (QIAGEN, China). 2 μg RNA was taken for reverse transcription into cDNA (M-MLV Reverse Transcriptase, Promega, USA) according to the manufacturer's instructions. qPCR was performed using sense (S) and antisense (AS) primers, 2 x SYBR Green qPCR Master Mix (Bimake.cn #B21202) reagent, cDNA and realplex2 qPCR system (Eppendorf, Germany). Refer to Supplementary Table S2 for detailed primer information.

**Cell viability, colony formation, and wound healing assays**

Cells (2×10^5 cells/well) were seeded in 24-well plates for 12 h and treated with different concentrations of peptides. After processing time, viable cells were counted by trypan blue staining (0.4% w/v). 3 Minutes later, cells incubated with the staining solution were washed and fixed at 4% paraformaldehyde for imaging. The number of viable cells was counted by ImageJ software to calculate the cell viability of each well. The relative cell viability versus the concentration of peptide was plotted and IC50 (μM) was calculated by GraphPad software.

Cells were seeded in a 6-well plate (200 cells/well) and incubated for 12 h at 37°C, followed by M1-21mut or M1-21 treatments, and replaced every three days with fresh medium containing peptides. After incubation for 14 days, the cells were fixed with 4% paraformaldehyde (w/v), rinsed three times with 1×PBS, stained with 0.1% crystal violet (w/v) for 10 min at room temperature, and finally washed three times with 1×PBS. Pictures of the colonies were taken with a digital camera.

Cells (1×10^6 cells/well) were seeded in 6-well plates. When the cells reached 90% confluence, a line was drawn with a 200 μl pipette tip and photos were taken and recorded as the 0 h group. Cells were then treated with M1-21mut or M1-21 and cell migration was recorded at 36 hours after wound formation. According to the imaging results, the cell migration area was calculated by ImageJ software, and the cell migration rate was calculated by GraphPad software. Migration rate % = [(Area 0 h - Area 36 h)/(Area 0 h)] × 100%.

**Pulldown and Co-immunoprecipitation Assays**

Biotinylated M1-21 was incubated with GST-tagged recombinant proteins or cell lysates for 3 hours at 4°C, followed by the addition of 20 μl Streptavidin Magnetic Beads (Beyotime, China) and further incubation for 1 hour. The magnetic beads were rinsed 5 times with pre-cooled 1×PBS, then the protein loading buffer was added, and the proteins were denatured at 95°C for 10 minutes. Finally, SDS-PAGE gel electrophoresis and Western blotting were performed.

For Co-IP, IP lysate buffer was used to extract total protein. 500 μg protein lysates were incubated with 20 μl of Anti-Flag Magnetic Beads (Bimake, USA) for Flag-FOXM1 proteins at 4 °C for 3 h. The beads were washed 5 times with pre-cooled 1×PBS and subjected to Western blotting.

**Luciferase activity assays**

HEK-293T cells (1×10^5 cells/well in 12-well plates) were transfected (EZ Trans, LIFE iLAB BIO, China) with certain luciferase reporter vectors (0.4 μg) and expression vector (0.6 μg) (or control plasmid (0.6 μg)). pRL-CMV plasmid (20 ng) was used as loading control for each transfection. 8 Hours after transfection, the medium was replaced and inhibitory peptides (or control peptide) with different concentrations were added. Finally, luciferase activity was measured 48 hours later with the Dual luciferase Assay system (Promega, USA) according to the manufacturer's instructions.

**Electrophoretic mobility shift assays (EMSAs)**

The double-strand DNA (dsDNA) probe was synthesized by Sangon Co., Ltd. (Shanghai, China). Protein (2 μM) was incubated with FAM-labeled dsDNA probe (50 nM) in binding buffer (20 mM Tris-Cl, 50 mM KCl, 0.5 mM EDTA, 0.2 mM DTT, 10% glycerol, pH 7.6) for 30 min on ice. Dosages of unlabeled cold probe (5 μM) or M1-21 of increased dose (0.5, 1, and 2 μM) for competitive or interaction experiments were added to reactions. The reactions were resolved in 4% native polyacrylamide gel electrophoresis in 0.5×TBE and visualized with Kodak 4000 MM Imaging System (Kodak, USA) (EX: 465 nm, EM: 535 nm for FAM). Sequence information is available in Supplementary Table S3.

**Immunohistochemistry**

Tumor tissues isolated from mice were first fixed with 4% paraformaldehyde, then dehydrated and embedded in paraffin for 2 days. Tumor sections (4 μm) were first dewaxed and rehydrated, followed by endogenous peroxidase quenching, antigen extraction (saline sodium citrate, microwave), and nonspecific binding site blocking. The sections were incubated with anti-KI-67 (1:200, Beyotime AF1738), anti-Foxm1 (1:200, Santa Cruz SC-376471), or anti-CDC25B (1:200, Santa Cruz SC-6948), rinsed with nonspecific binding antibody, and then incubated with horseradish peroxidase conjugated secondary antibody. The color was detected with 3,3'-diaminobenzidine and photographed at a Nikon TE2000 (Nikon, Japan).

**References**

1. Xie ZQ, Tan GX, Ding MA, Dong DF, Chen TH, Meng XX, et al. Foxm1 transcription factor is required for maintenance of pluripotency of P19 embryonal carcinoma cells. Nucleic Acids Res. 2010;38(22):8027-38.

2. Xiang Q, Tan G, Jiang X, Wu K, Tan W, and Tan Y. Suppression of FOXM1 Transcriptional Activities via a Single-Stranded DNA Aptamer Generated by SELEX. Sci Rep. 2017;7(45377.

3. Zhang Z, Bu H, Yu J, Chen Y, Pei C, Yu L, et al. The cell-penetrating FOXM1 N-terminus (M1-138) demonstrates potent inhibitory effects on cancer cells by targeting FOXM1 and FOXM1-interacting factor SMAD3. Theranostics. 2019;9(10):2882-96.

**Supplementary Table S1.**

| **REAGENT or RESOURCE** | **SOURCE** | **USAGE** | **IDENTIFIER** |
| --- | --- | --- | --- |
| **Antibodies and Purification Beads** | | | |
| Rabbit monoclonal anti-FOXM1 (clone D3F2B) | Cell Signaling Technology | 1:1000 (WB) | Cat# 20459S |
| Mouse monoclonal anti-FOXM1 (clone G-5) | Santa Cruz | 1:500 (WB) | Cat# sc-376471 |
| Anti-Flag Magnetic Beads | Bimake | 20 μL (Each hole) | Cat# B26102 |
| BeyoMag™ Streptavidin Magnetic Beads | Beyotime | 20 μL (Each hole) | Cat# P2151-5mL |
| Rabbit monoclonal anti-DYKDDDDK Tag (clone D6W5B) | Cell Signaling Technology | 1:1000 (WB) | Cat# 14793 |
| Rabbit polyclonal anti-FLAG Tag | Beyotime | 1:2000 (WB) | Cat# AF0036 |
| Streptavidin-HRP | Beyotime | 1:5000 (WB) | Cat# A0303 |
| Rabbit polyclonal anti-β-Actin (D8) | Bioworld Technology | 1:10000 (WB) | Cat# AP0731 |
| Rabbit monoclonal anti-Lamin B1 | Beyotime | 1:1000 (WB) | Cat# AF1408 |
| Mouse monoclonal anti-β-Tubulin | Beyotime | 1:1000 (WB) | Cat# AF2835 |
| Rabbit polyclonal anti-PLK1 | Bioworld Technology | 1:1000 (WB) | Cat# BS7198 |
| PLK1 Rabbit Monoclonal Antibody | Beyotime | 1:1000 (WB) | Cat# AG2902 |
| Mouse polyclonal anti-Cdc25B | Abcam | 1:2000 (WB) | Cat# ab167347 |
| Rabbit polyclonal anti-CDC25B(E-19) | Santa Cruz | 1:100 (IHC) | Cat# sc-6948 |
| Rabbit polyclonal anti-PCNA | Sangon Biotech | 1:2000 (WB) | Cat# D220014 |
| Rabbit monoclonal anti-Cyclin B1 | Beyotime | 1:2000 (WB) | Cat# AF1606 |
| Rabbit monoclonal anti-Vimentin | Beyotime | 1:2000 (WB) | Cat# AF1975 |
| Rabbit polyclonal anti-N-Cadherin | Cell Signaling Technology | 1:1000 (WB) | Cat# 13116 |
| Rabbit polyclonal anti-E-Cadherin | Cell Signaling Technology | 1:1000 (WB) | Cat# 3195S |
| Mouse monoclonal anti-LIN9(C-10) | Santa Cruz | 1:500 (WB) | Cat# sc-398234 |
| Mouse monoclonal anti-B-MYB(C-5) | Santa Cruz | 1:500 (WB) | Cat# sc-390198 |
| Mouse monoclonal anti-β-catenin | BD Biosciences | 1:2000 (WB) | Cat# 610153 |
| Rabbit Monoclonal Anti-Ki-67 | Beyotime | 1:200 (IHC) | Cat# AF1738 |
| Horseradish peroxidase-conjugated goat anti-rabbit IgG(H+L) | Beyotime | 1:2000 (WB) | Cat# A0208 |
| Horseradish peroxidase-conjugated goat anti-mouse IgG(H+L) | Beyotime | 1:2000 (WB) | Cat# A0216 |
| BeyoGold™ His-tag Purification Resin | Beyotime | 200 μL (Each sample) | Cat# P2218 |
| BeyoGold™ GST-tag Purification Resin | Beyotime | 200 μL (Each sample) | Cat# P2255 |
| **Bacterial and virus strains** | | | |
| DH5α Competent Cells | Sangon Biotech | Cat# B528413 | |
| Stbl3 Competent Cells | Lab Homemade |  | |
| BL21(DE3) Competent Cells | Sangon Biotech | Cat# B528414 | |
| Lentivirus system | Lab Homemade |  | |
|  |  |  | |
| **Reagents and Kits** | | | |
| Endotoxin-Free Plasmid Mini Extraction Kit | TIANGEN | Cat# DP118-02 | |
| EZ Trans | LIFE iLAB BIO | Cat# AC04L092 | |
| Blood/Cell/Tissue Genomic DNA Extraction Kit | TIANGEN | Cat# DP304-03 | |
| M-MLV Reverse Transcriptase | Promega | Cat# M1701 | |
| In-Fusion Snap Assembly Master Mix | TaKaRa | Cat# 638947 | |
| Dual-Luciferase® Reporter Assay System | Promega | Cat# E1960 | |
| Puromycin Dihydrochloride | Beyotime | Cat# ST551-50mg | |
| Doxycycline Hyclate | Beyotime | Cat# ST039A | |
| D-Luciferin potassium salt | Beyotime | Cat# ST196 | |
| NHS-Biotin | Sangon Biotech | Cat# C100212-0050 | |
| NHS-ICG | XIAN QIYUE BIOLOGY | Cat# 1622335-40-3 | |
| Hematoxylin and Eosin Staining Kit | Beyotime | Cat# C0105M | |
| Amino acids and resins for synthesis | CS Bio | http://www.csbiochina.com | |

**Supplementary Table S2.**

| **RT-PCR Primers** | **5’ - 3’** |
| --- | --- |
| FOXM1-Forward | GCTTGCCAGAGTCCTTTTTGC |
| FOXM1-Reverse | CCACCTGAGTTCTCGTCAATGC |
| PLK1-Forward | CCTGCACCTCAGCAACGGCA |
| PLK1-Reverse | CCATAGTGCGGGCGTAGCGG |
| CDC25B-Forward | AGTCCTGACCGGAAGATGGA |
| CDC25B-Reverse | GATGTTGCTGAACTTGCCCG |
| PCNA-Forward | ACTAACTTTTGCACTGAGGTACC |
| PCNA-Reverse | GTATTTTAAGTGTCCCATATCCGC |
| CCNB1-Forward | GGTCTGGGTCGGCCTCTACCT |
| CCNB1-Reverse | AGCCAGGTGCTGCATAACTGGAA |
| Vimentin-Forward | GAGAACTTTGCCGTTGAAGC |
| Vimentin-Reverse | GCTTCCTGTAGGTGGCAATC |
| N-cadherin-Forward | GACGGTTCGCCATCCAGA C |
| N-cadherin-Reverse | TCGATTGGTTTGACCACGG |
| E-cadherin-Forward | CGGGAATGCAGTTGAGGATC |
| E-cadherin-Reverse | AGGATGGTGTAAGCGATGGC |
| GAPDH-Forward | GGAGCGAGATCCCTCCAAAAT |
| GAPDH-Reverse | GGCTGTTGTCATACTTCTCATGG |

**Supplementary Table S3.**

| **EMSAs DNA probe** | **5’ - 3’** | **Modified state** |
| --- | --- | --- |
| dsDNA probe forward strand | FAM-TTTGTTTATTTGTTTGTTTATTTG | FAM |
| dsDNA probe reverse strand | CAAATAAACAAACAAATAAACAAA | NO |
| Cold dsDNA probe forward strand | TTTGTTTATTTGTTTGTTTATTTG | NO |
| Cold dsDNA probe reverse strand | CAAATAAACAAACAAATAAACAAA | NO |

**Original WB data**

**Figure 1D**


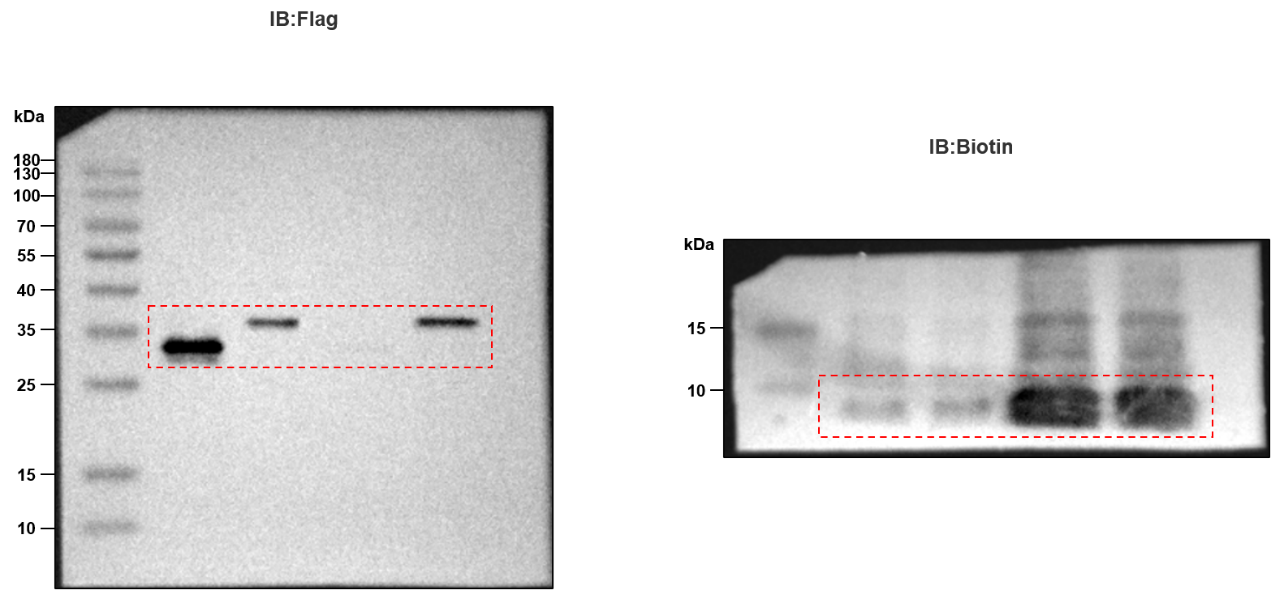


**Figure 1E**


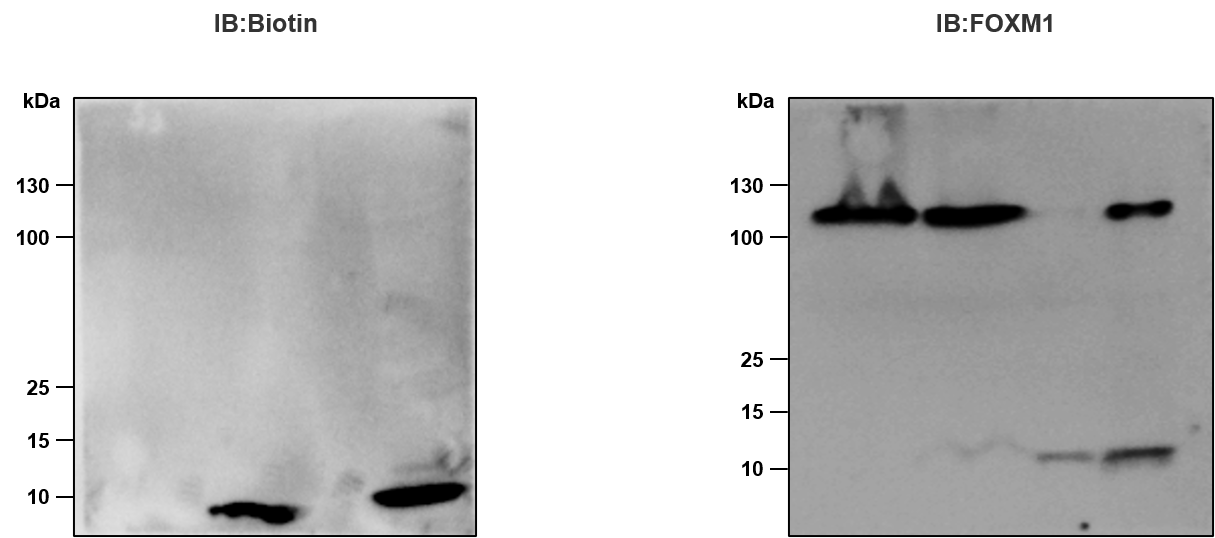


**Figure 2B**


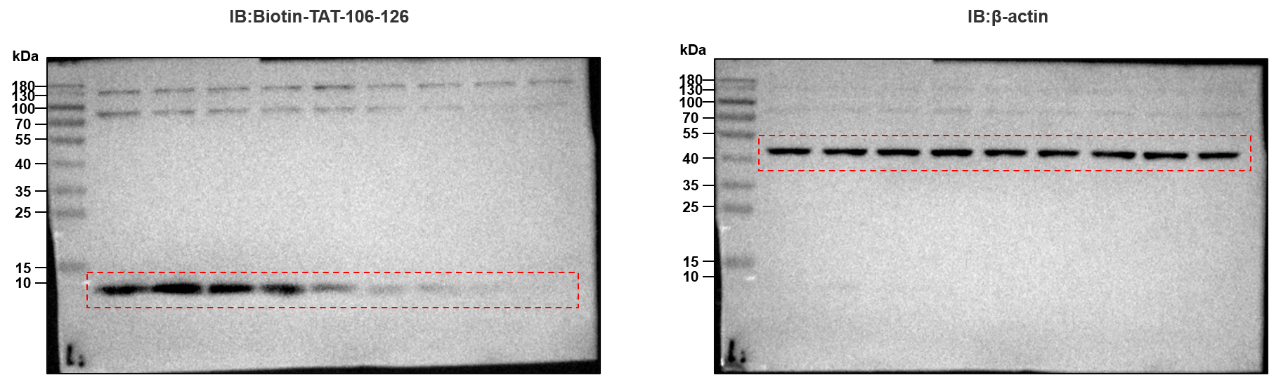


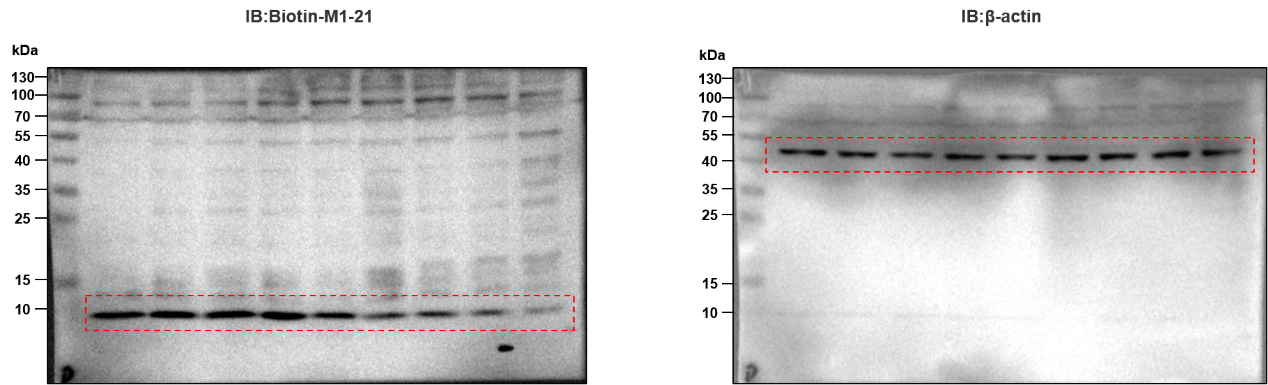


**Figure 2E**


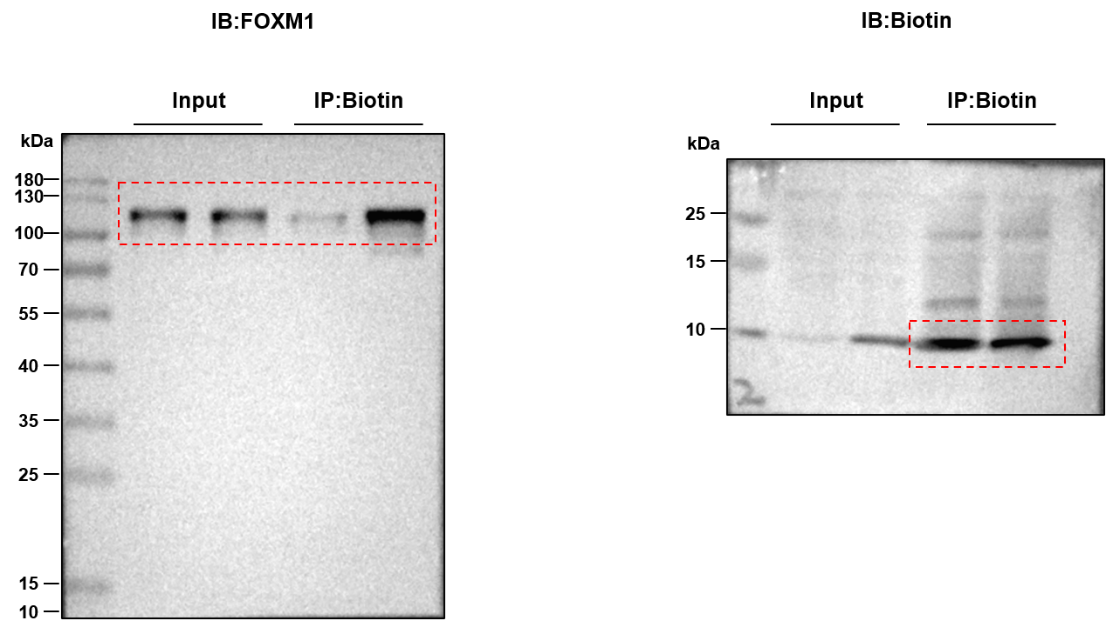


**Figure 2F**


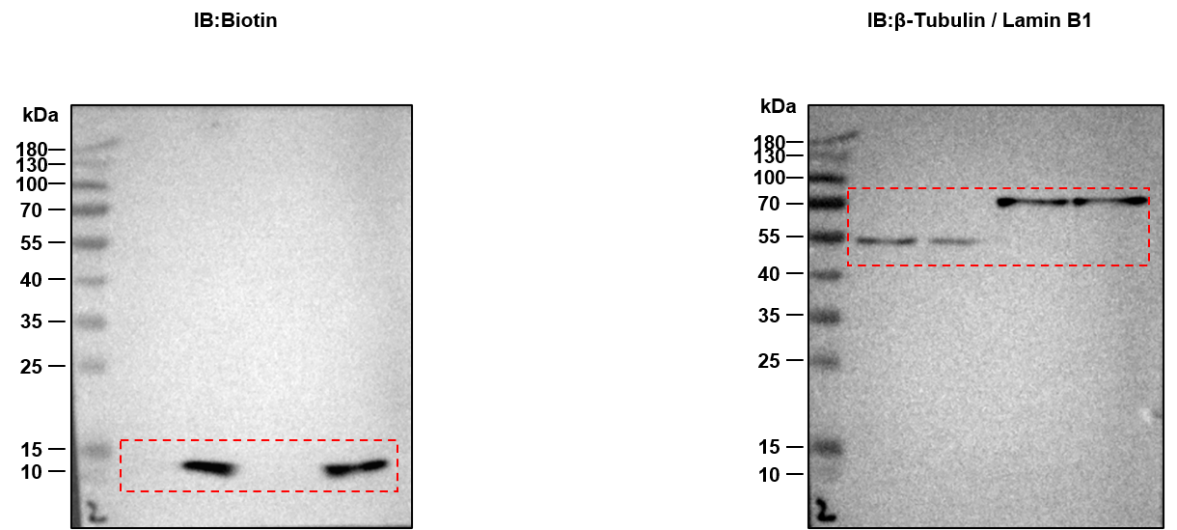


**Figure 3D**


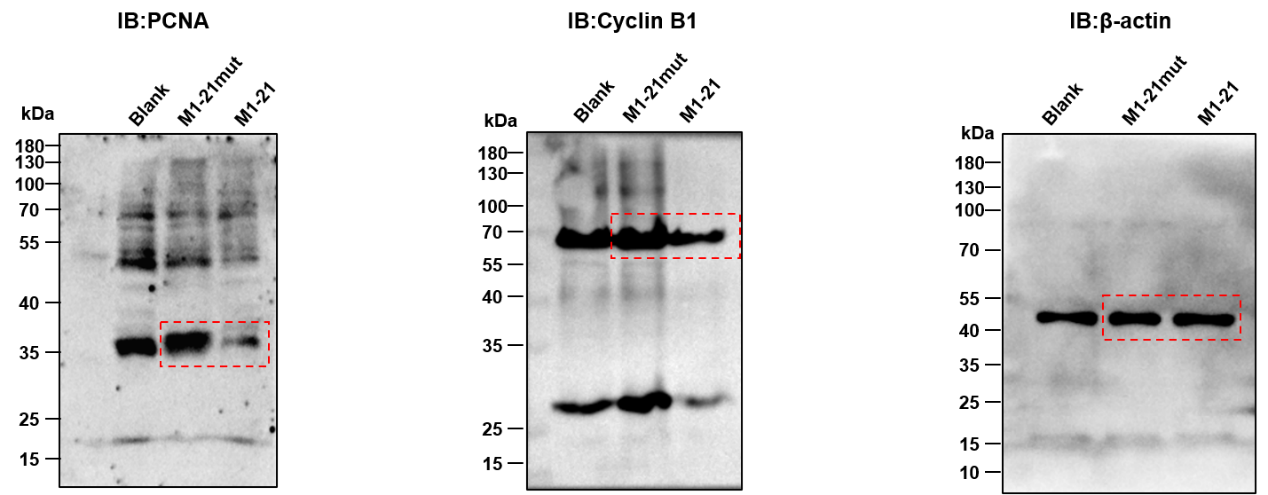


**Figure 3J**


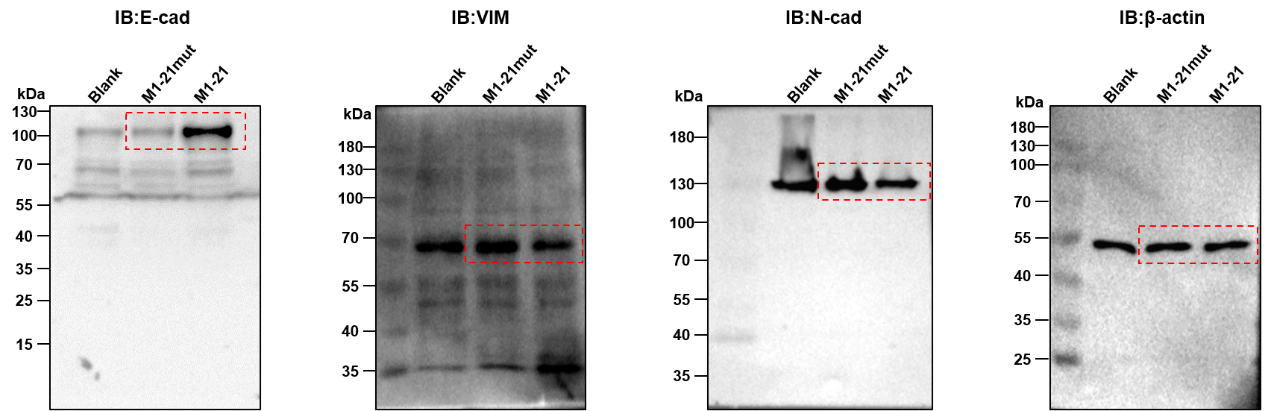


**Figure 4A**


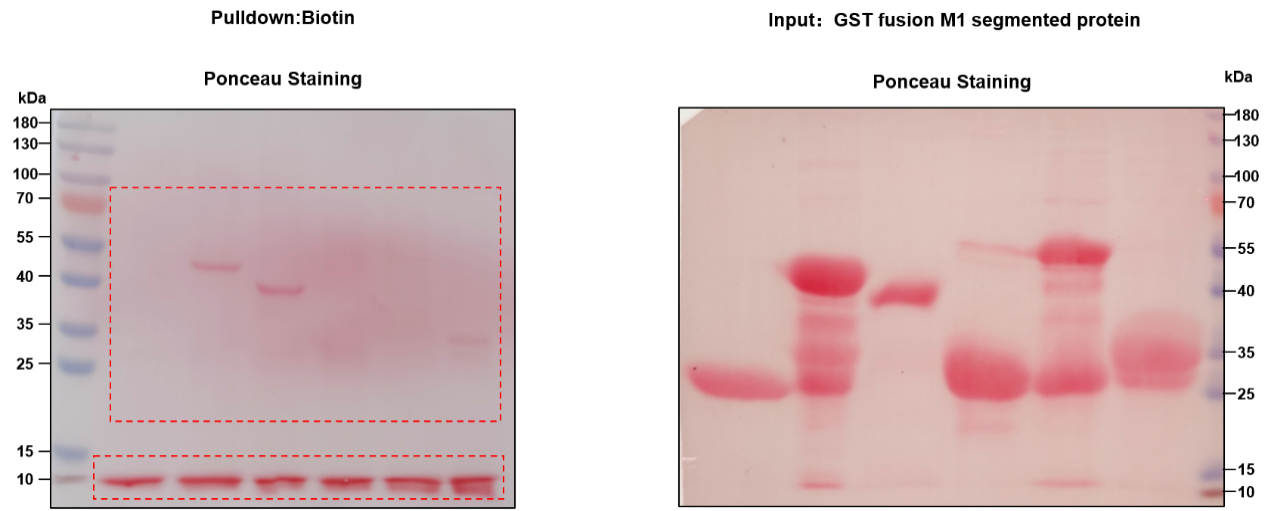


**Figure 4D**


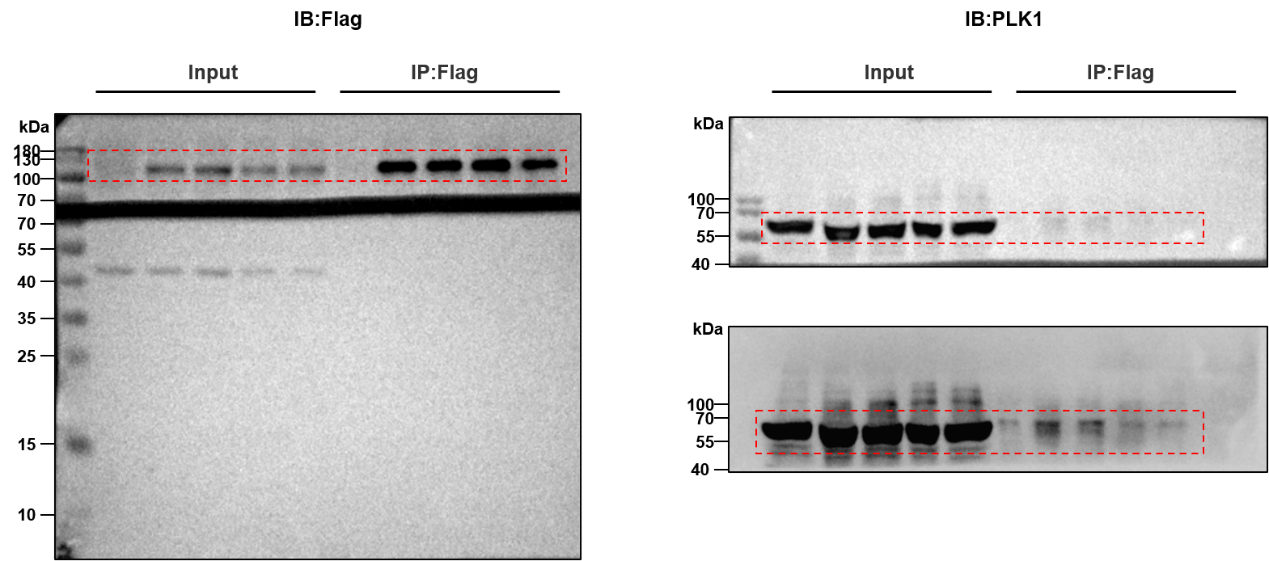


**Figure 4E**


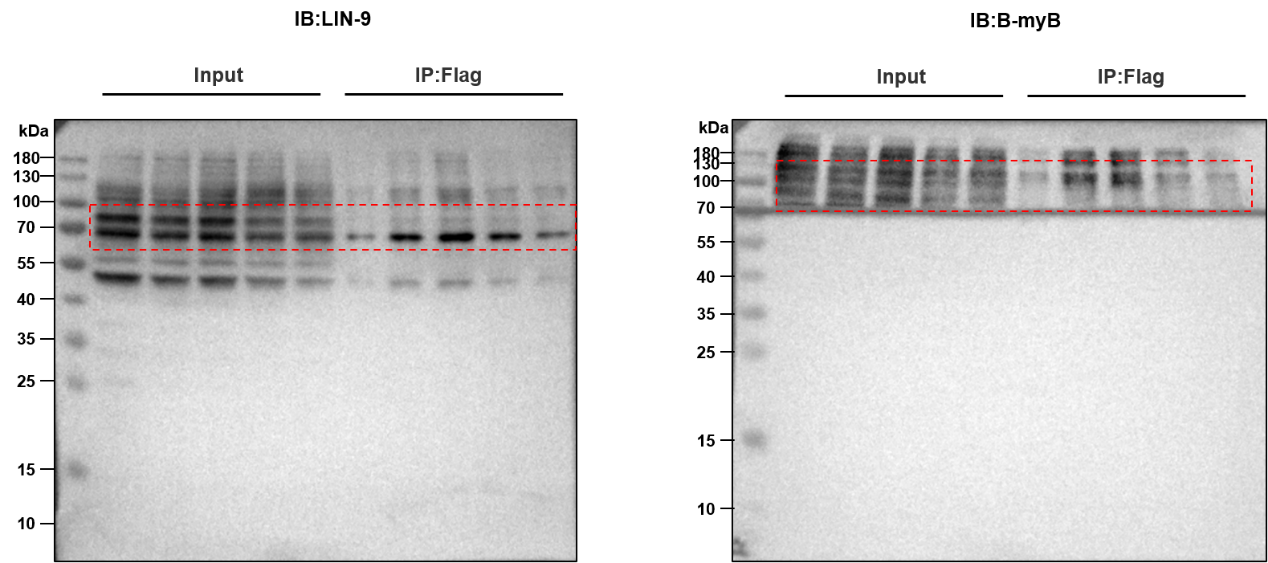


**Figure 4G**


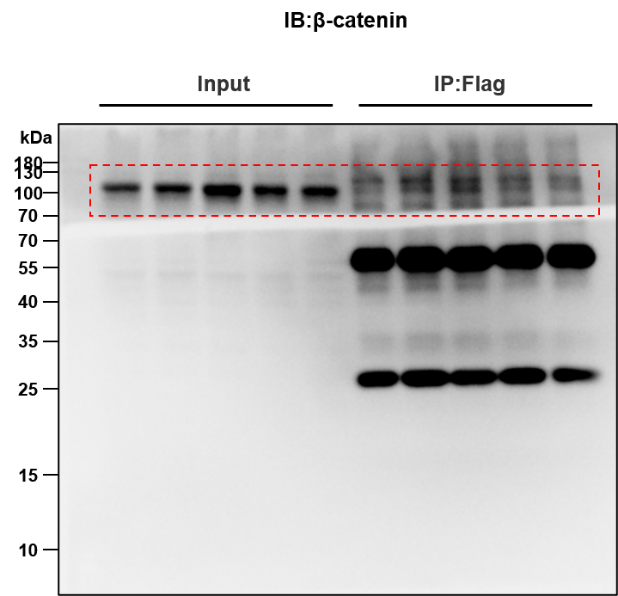


**Figure 4H**


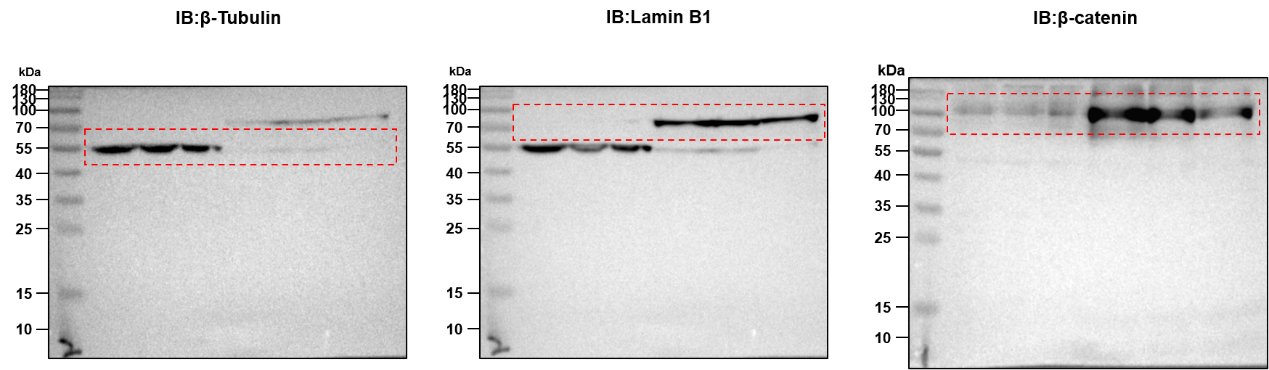


**Figure 4K**


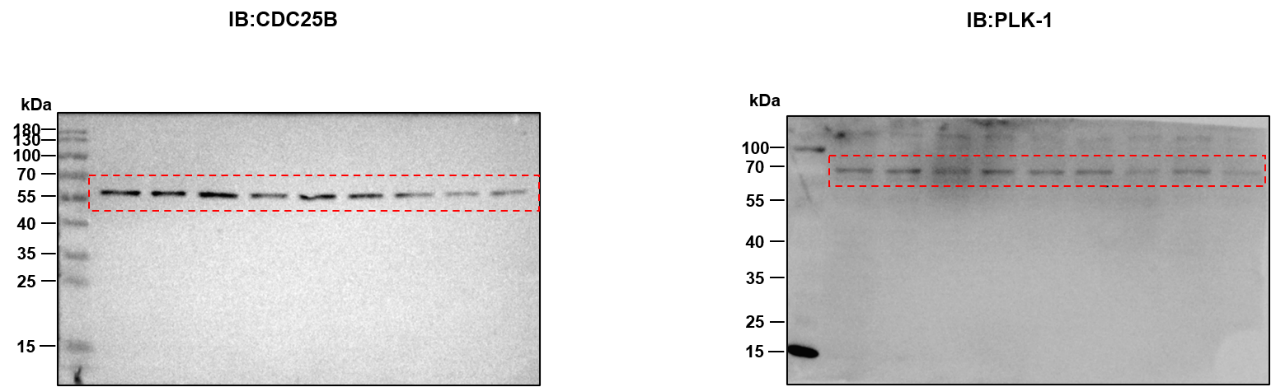


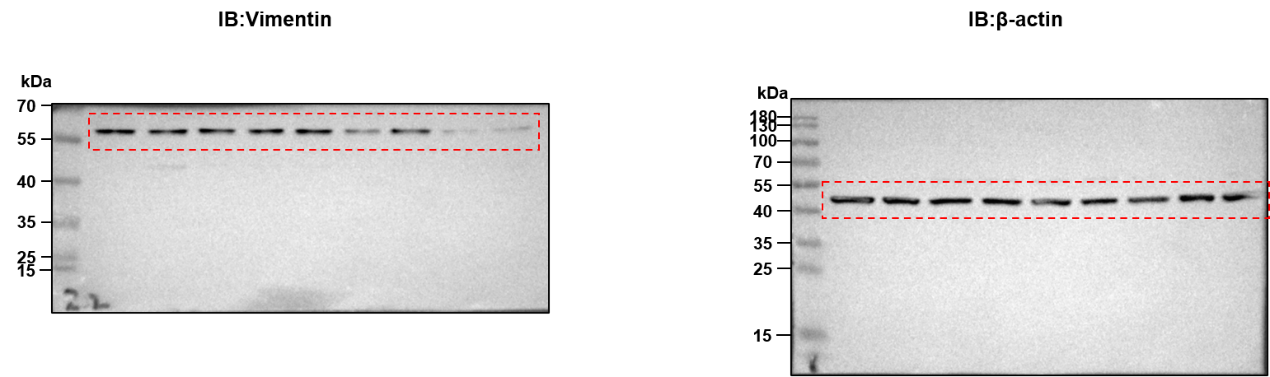


**Figure S5A**


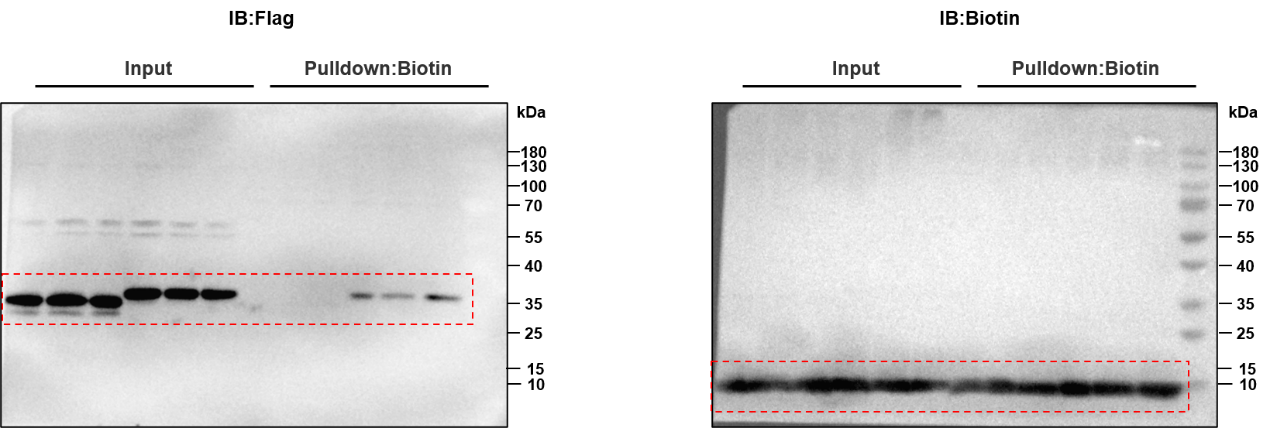


**Figure S5B**


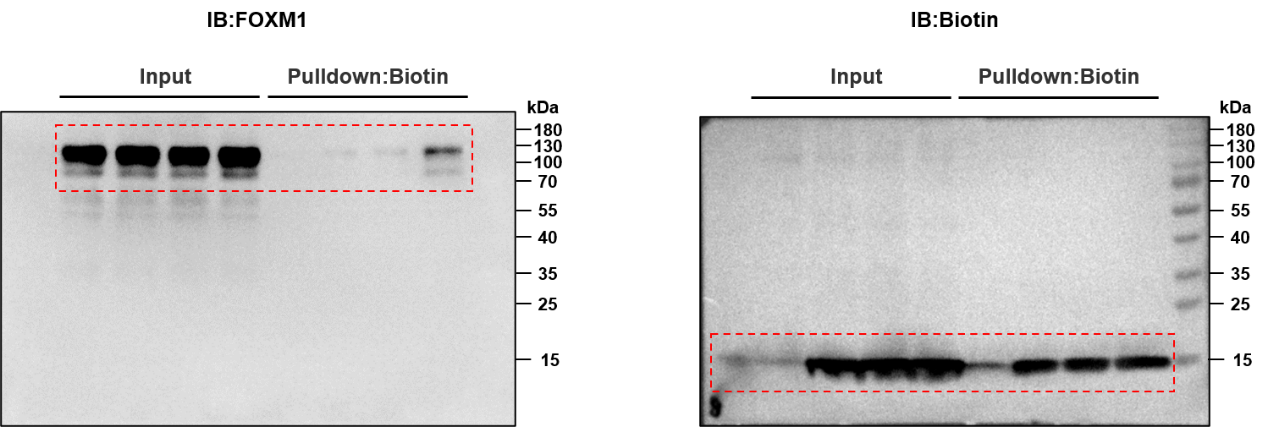


**Figure S12B**


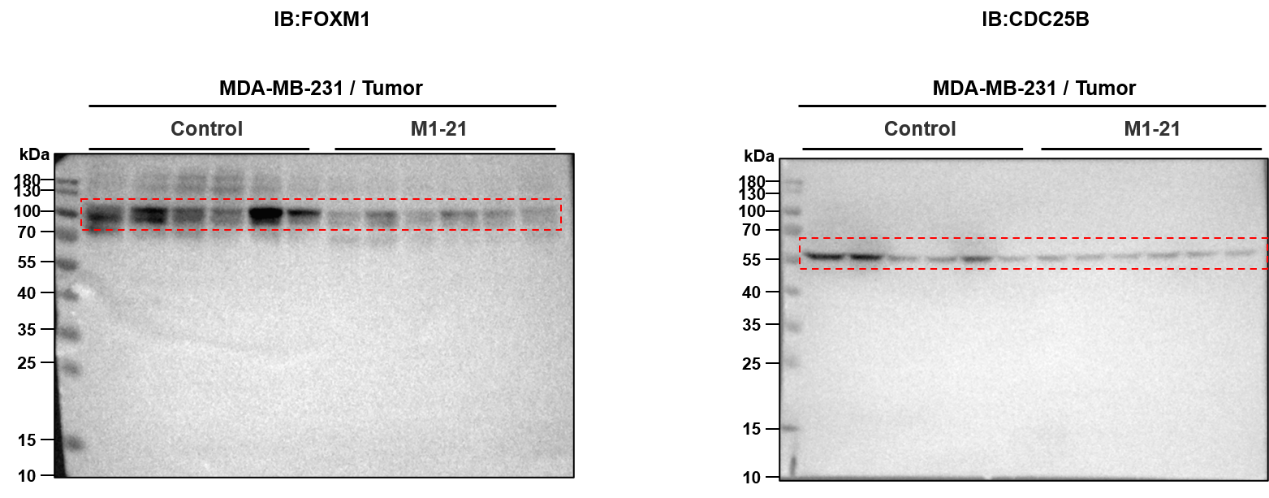


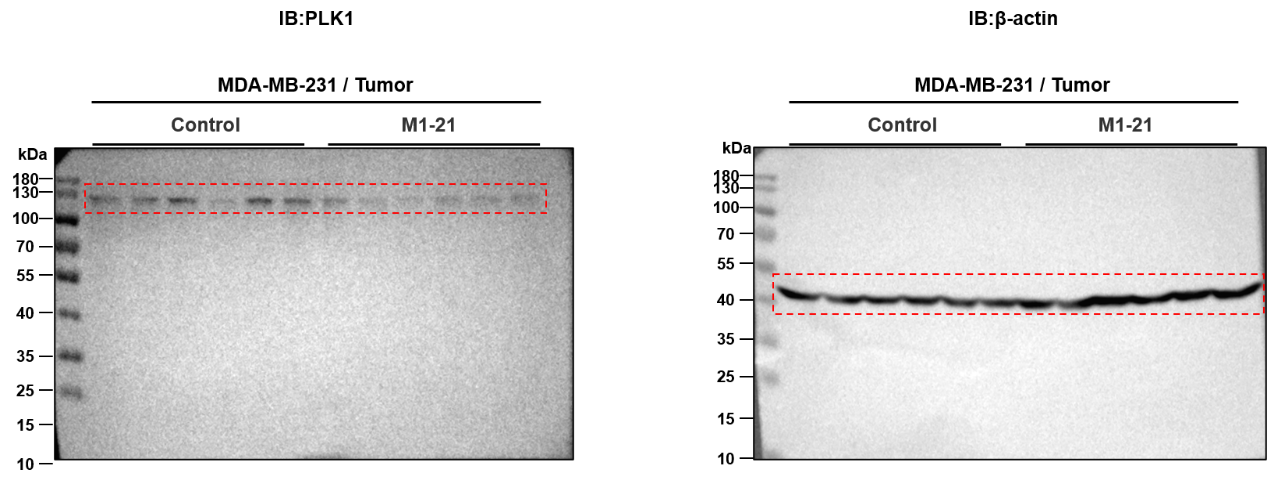


**Figure S15A**


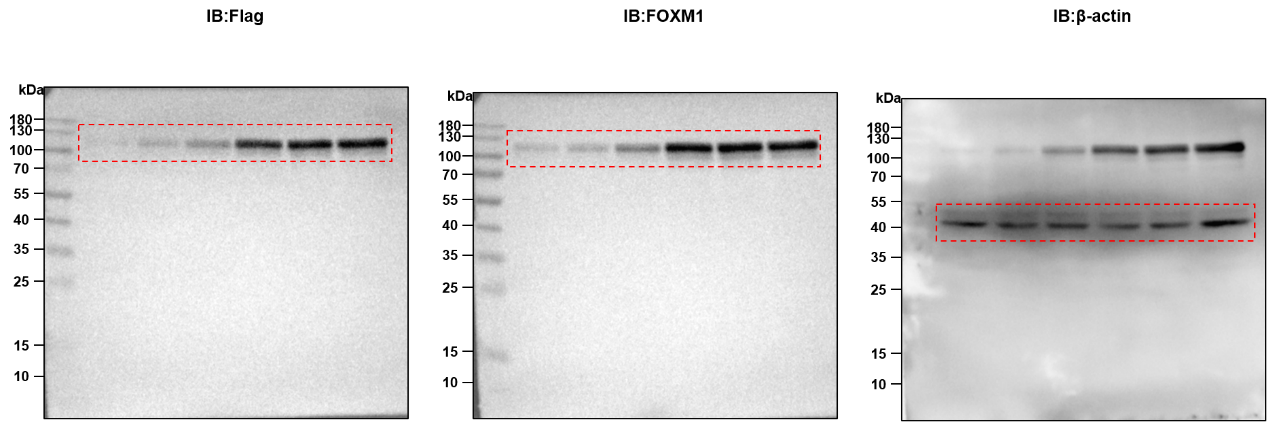


**Figure S15B**


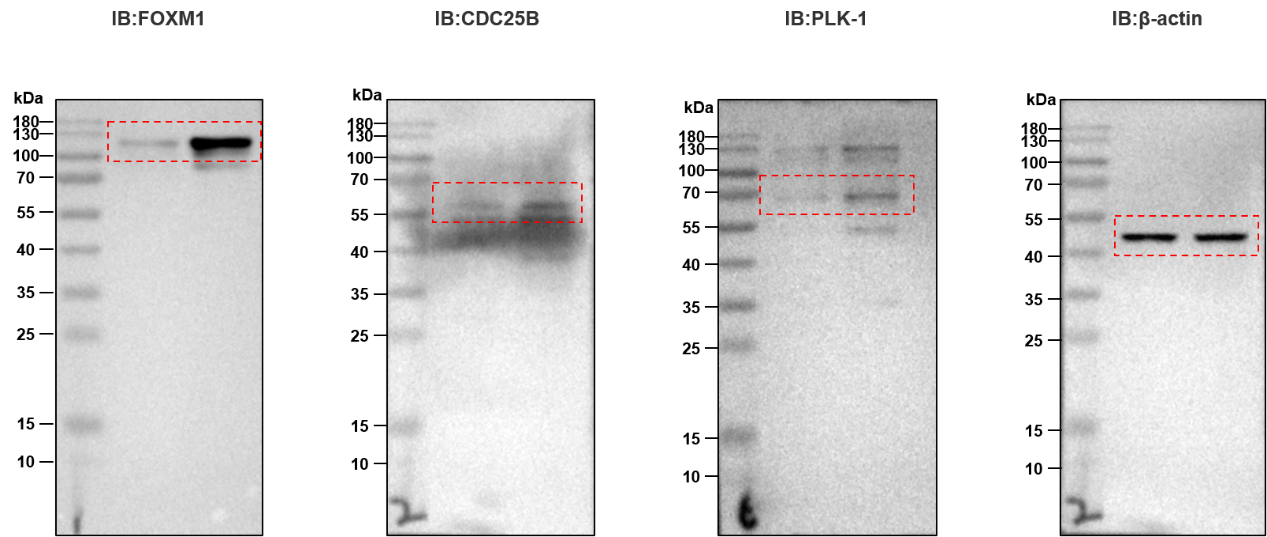


**Figure S16**


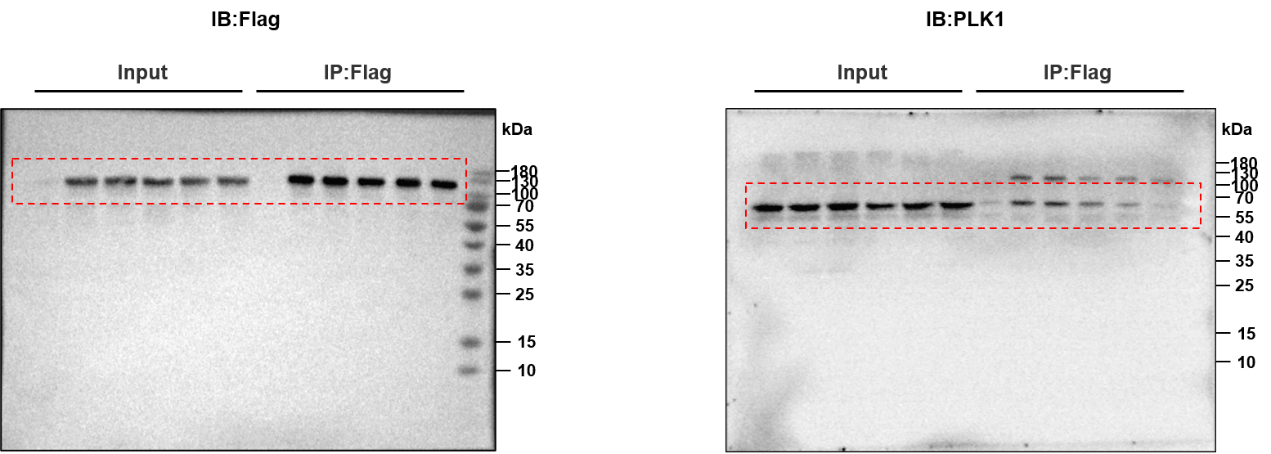


**Figure S18B**


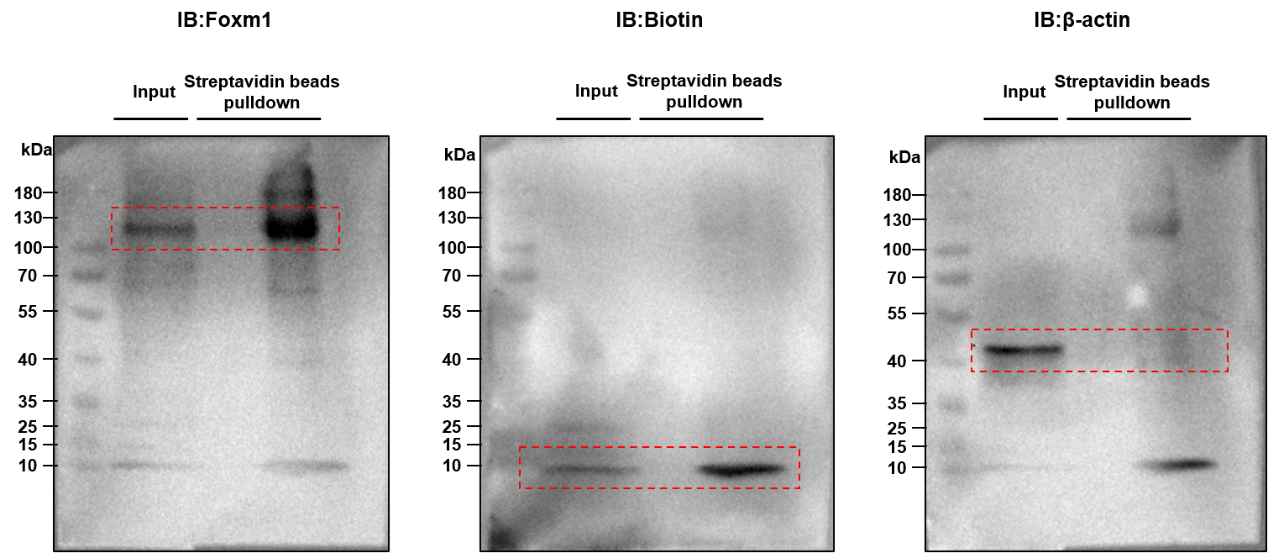


**Figure S21B**


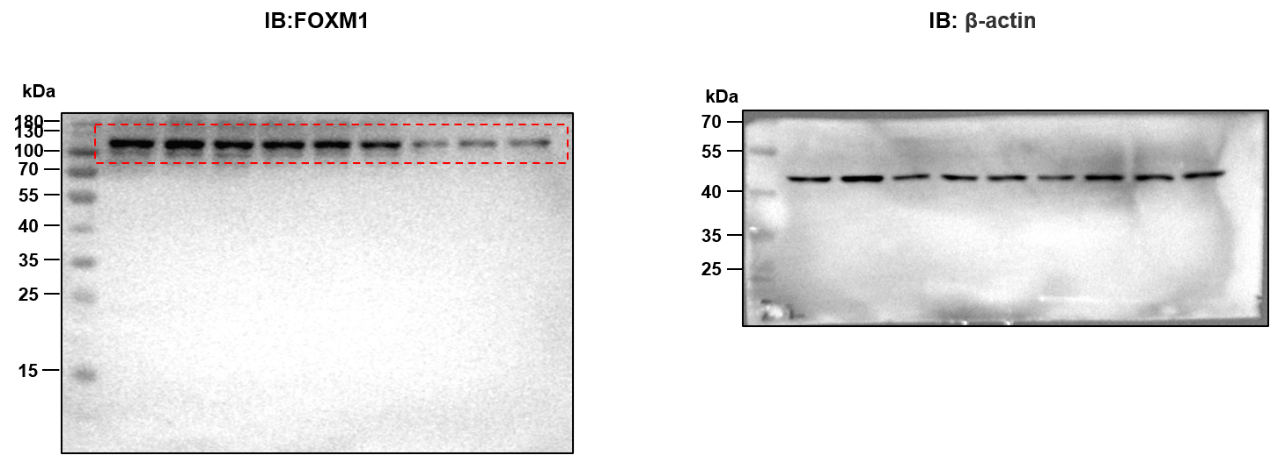

Supplement: Supplementary file 1 — Supplementary Material 1 [file 13578_2023_1059_MOESM1_ESM.docx]
